# Fitness Distance Balanced Starfish Optimization for Benchmark and Engineering Design Problems

**DOI:** 10.3390/biomimetics11060390

**Published:** 2026-06-02

**Authors:** Tuğrul Yağbasan, Ömür Akyazı, Hayati Türe, Bekir Dizdaroğlu

**Affiliations:** 1 Department of Computer Engineering, Karadeniz Technical University, Trabzon 61080, Türkiye; bekir@ktu.edu.tr; 2 Department of Energy Systems Engineering, Karadeniz Technical University, Trabzon 61080, Türkiye; 3 Department of Computer Engineering, Trabzon University, Trabzon 61080, Türkiye; hayatiture@trabzon.edu.tr

**Keywords:** biomimetic optimization, starfish optimization, diversity-aware selection, fitness–distance balance, engineering design optimization, bio-inspired computation

## Abstract

Biomimetic optimizers are increasingly used to solve complex engineering problems, yet their performance depends strongly on how effectively they preserve diversity while maintaining selection pressure toward promising regions. In this study, the Starfish Optimization Algorithm (SFOA) is enhanced through fitness–distance-aware selection control, leading to two improved variants: Fitness–Distance Balance Starfish Optimization Algorithm (FDBSFOA) and Dynamic Fitness–Distance Balance Starfish Optimization Algorithm (dFDBSFOA). The proposed framework guides candidate selection using both solution quality and spatial diversity relative to the current best solution, while the dynamic variant further adapts this balance over the course of the search to improve exploration in early iterations and exploitation near convergence. The proposed methods are evaluated on the IEEE CEC2017, CEC2020, and CEC2022 benchmark suites under a unified maximum function evaluation budget, *MaxFEs* = 10,000 × *D*, with 21 independent runs, and are further validated on constrained engineering design problems. Performance is assessed using convergence behavior, robustness indicators, computational overhead, and nonparametric statistical tests. The results show that the proposed variants improve the robustness and search efficiency of baseline SFOA, with dFDBSFOA providing the most consistent overall performance while introducing a controlled and interpretable computational overhead. These findings suggest that diversity-aware selection can serve as an effective design principle for strengthening biomimetic optimization frameworks. The current study focuses mainly on continuous, single-objective, and stationary benchmark problems, while the engineering-design validation also includes constrained and discrete/integer-coded cases. Extending the proposed strategy to dynamic, noisy, large-scale mixed-integer, or multi-objective settings remains future work.

## 1. Introduction

Biological systems have long inspired computational search strategies because they exhibit adaptive, decentralized, and resilient behaviors under uncertain conditions. In this context, biomimetic optimization has become an important research direction for solving complex engineering and computational problems. From a practical standpoint, however, the success of a biomimetic optimizer depends not only on the natural metaphor underlying its search operators, but also on how effectively the search process preserves informative diversity while maintaining sufficient selection pressure toward promising regions.

Optimization is a fundamental problem-solving paradigm widely adopted in engineering, artificial intelligence, and computational science [1,2,3,4]. In general, optimization aims to identify the best feasible solution within a bounded search space under a set of constraints. However, many real-world problems are nonlinear, nonconvex, multimodal, and high-dimensional; therefore, classical deterministic and gradient-based techniques can be sensitive to initialization and may stagnate in locally optimal regions [5,6,7]. To address these limitations, metaheuristic optimization algorithms (MOAs) have become practical alternatives due to their derivative-free nature and global search capability across complex landscapes [8,9,10]. Representative examples include Genetic Algorithm (GA) [11], Particle Swarm Optimization (PSO) [12], Differential Evolution (DE) [13], Teaching–Learning-Based Optimization (TLBO) [14], Grey Wolf Optimizer (GWO) [15], Equilibrium Optimizer (EO) [16], and Harris Hawks Optimization (HHO) [17]. Although these methods are inspired by different principles and may show different empirical behaviors, maintaining a robust exploration–exploitation balance remains a persistent challenge for locating high-quality solutions, especially on rugged and high-dimensional landscapes [8]. In practice, this balance is strongly coupled with selection pressure and diversity preservation; overly aggressive exploitation may collapse diversity and trigger premature stagnation, whereas excessive exploration can delay refinement [18,19]. This motivates selection-driven enhancement mechanisms that explicitly account for both solution quality and spatial distribution, such as fitness–distance-aware strategies [19,20,21]. Since MOAs rely on randomized components, outcomes can vary across runs; therefore, repeated trials and statistically sound comparisons are commonly adopted when evaluating algorithms on standardized benchmark protocols [22,23,24].

Among recent biomimetic optimizers, the Starfish Optimization Algorithm (SFOA) is a population-based method that models starfish behaviors such as exploration, preying, and regeneration [25]. Despite its promising performance, like many swarm-type methods, SFOA may still experience premature convergence or loss of diversity on complex landscapes and at higher dimensions. This limitation is particularly important from a biomimetic optimization perspective, because biologically inspired movement operators may lose effectiveness when candidate guidance becomes overly fitness-driven or when the population collapses prematurely toward narrow regions of the search space.

The key gap addressed in this paper is that fitness–distance-aware selection principles, which promote informative diversity while retaining competitiveness, have not been systematically embedded into the SFOA search lifecycle and validated under a broad, standardized evaluation protocol. Therefore, an SFOA-based enhancement is needed that (i) accounts for spatial distribution relative to the current best solution to preserve diversity [18] and (ii) adaptively regulates selection pressure via fitness–distance-aware logic throughout the run [20,21].

Accordingly, this study proposes two enhanced variants, namely Fitness–Distance Balance Starfish Optimization Algorithm (FDBSFOA) and Dynamic Fitness–Distance Balance Starfish Optimization Algorithm (dFDBSFOA), and evaluates them on IEEE CEC benchmark suites and constrained engineering design problems to examine accuracy, robustness, and convergence behavior.

The novelty of this work lies not in introducing a new biological metaphor, but in redesigning the internal selection dynamics of a biomimetic optimizer. More specifically, the study shows that starfish-inspired search can be strengthened by integrating fitness–distance-aware candidate guidance into the SFOA search lifecycle, and that this guidance becomes more effective when the fitness–diversity trade-off is dynamically scheduled throughout the search. In this sense, the proposed framework contributes not merely an incremental performance modification, but a transferable design strategy for improving biomimetic optimization systems through diversity-aware selection control.

The main contributions of this work are summarized as follows:

- **FDBSFOA:** We integrate the Fitness–Distance Balance (FDB) selection mechanism into SFOA to strengthen biomimetic search through diversity-aware candidate guidance rather than purely fitness-driven pressure [20,25].
- **dFDBSFOA:** We introduce a dynamic variant based on dFDB to adapt the fitness–distance trade-off over iterations and improve the exploration–exploitation transition of the starfish-inspired search process [21].
- **Comprehensive validation:** We provide extensive benchmarking on CEC test suites and classical constrained engineering design problems under consistent settings.
- **Statistical verification:** We report rigorous nonparametric statistical analyses to support the significance of observed performance differences [22,23,24].
- **Cost awareness:** We discuss computational overhead and runtime behavior to show that the proposed biomimetic enhancements are obtained without disproportionate computational cost.

**Figure 1:**
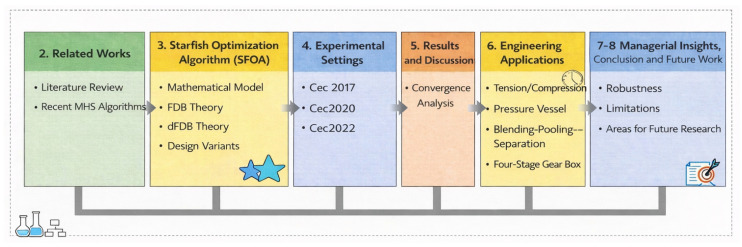
The structure and organization of the remaining sections are summarized.

The overall organization of the remainder of this paper is illustrated in Figure 1.

Metaheuristic optimization algorithms (MOAs) have attracted sustained research interest because of their derivative-free search capability and their flexibility in handling nonlinear, nonconvex, multimodal, and high-dimensional optimization problems [8]. In the literature, MOAs are commonly classified into major families according to their source of inspiration and update mechanism. Evolutionary algorithms improve candidate solutions through variation and selection operators, with Genetic Algorithm (GA) [11] and Differential Evolution (DE) [13] being among the most representative examples. Swarm-intelligence-based algorithms rely on collective learning and interaction rules, as illustrated by Particle Swarm Optimization (PSO) [12], Grey Wolf Optimizer (GWO) [15], and Harris Hawks Optimization (HHO) [17]. Physics- or mathematics-inspired algorithms, in contrast, formulate the search process by modeling system dynamics, equilibrium states, or transition behavior, as exemplified by the Equilibrium Optimizer (EO) [16]. Human- or learning-inspired approaches mimic pedagogical or social learning processes; for instance, Teaching–Learning-Based Optimization (TLBO) updates solutions through teacher and learner phases while requiring only a limited number of control parameters [14]. More recently, the Starfish Optimization Algorithm (SFOA) has been introduced as a bio-inspired optimizer and benchmarked against a large set of competitors, further enriching the contemporary MOA literature [25]. Despite their diverse inspirations and search operators, the effectiveness of these algorithms is ultimately governed by how successfully they balance exploration and exploitation while preserving sufficient population diversity throughout the search process [8].

Beyond these classical frameworks, recent studies have increasingly focused on proposing new MOAs or enhancing existing ones through hybrid structures, adaptive parameter control, learning-assisted mechanisms, and application-oriented integration, followed by validation on benchmark suites and practical optimization problems [26,27,28,29,30,31,32]. Additional recent contributions further demonstrate the continuing expansion of MOA research, including the tornado optimizer with Coriolis force, the supercell thunderstorm algorithm, the lice-inspired optimization algorithm, the cuckoo catfish optimizer, and comparative benchmark studies on emerging metaheuristics [33,34,35,36,37]. Studies published in 2025, in particular, highlight three prominent directions in this evolving landscape. First, newly developed bio-inspired optimizers continue to broaden the methodological scope of MOAs, as evidenced by the Lionfish Search Algorithm and plant-inspired search models derived from water uptake and transport dynamics [26,27]. Second, established optimizers are being reinforced through hybrid and adaptive design strategies, such as crisscross-learning-enhanced snake optimization and reinforcement-learning-based improved snake optimization for real-world engineering problems [28,29]. Third, MOAs are increasingly embedded into application-specific optimization frameworks, including random walk-enhanced engineering optimizers, trust-aware MANET routing supported by hierarchical manta ray foraging optimization, and hybrid communication system models developed for PAPR reduction in UFMC/B5G settings [30,31,32]. Taken together, these studies indicate that contemporary MOA research is increasingly characterized not only by algorithmic novelty, but also by adaptive control, hybridization, and application-aware integration aimed at improving robustness, convergence characteristics, and real-world performance.

Another important methodological direction in recent MOA research concerns the design of selection and diversity-preservation mechanisms to stabilize performance and alleviate premature convergence. In population-based MOAs, selection determines which candidate solutions guide the search and how information is propagated across the population; accordingly, selection pressure has a direct influence on both convergence speed and diversity retention [8]. Excessively strong selection may rapidly reduce population diversity and drive the search toward stagnation, whereas excessively weak selection can delay exploitation and compromise final solution quality [8]. For this reason, diversity-aware strategies, often incorporating distance- or dispersion-based information, have been investigated to improve search-space coverage and to prevent early crowding around suboptimal regions [18]. Within this line of research, fitness–distance-aware selection principles have been introduced to jointly account for solution quality and spatial diversity during candidate ranking and survival. Notable examples include Fitness–Distance Balance (FDB) [20] and its dynamic extension, dFDB, which adjusts the fitness–distance trade-off over the course of the search [21]. Related efforts further suggest that selection pressure can be explicitly modulated within learning-based optimizers; for example, tournament-style TLBO has been reported as a mechanism for strengthening selection behavior in TLBO-driven optimization [38]. This perspective is especially relevant for biomimetic optimizers such as SFOA, where movement-inspired operators require effective internal candidate guidance to remain robust in complex search spaces.

Since MOAs are stochastic by nature, comparative evaluation has evolved toward more rigorous experimental protocols that emphasize repeated independent runs, fair computational budgets (e.g., MaxFEs), and statistical assessment for more reliable performance interpretation [37,38]. Accordingly, contemporary studies increasingly report not only numerical performance indicators but also comparative statistical evidence to support more robust conclusions across benchmark problems and practical optimization scenarios [37,38].

To facilitate a structured discussion, Table 1 maps the metaheuristics referenced in this section to their upper-level families, whereas Table 2 summarizes the publication year, methodological type, and main search principle of the key cited methods together with representative recent (2025) MOA-related studies.

## 2. Methodology

### 2.1. Starfish Optimization Algorithm

The Starfish Optimization Algorithm (SFOA) is a nature-inspired metaheuristic optimization method inspired by starfish behavior, with a focus on exploration, hunting, and regeneration. The basic operating structure of SFOA consists of two phases: exploration and exploitation. During the exploration phase, search effectiveness is improved by combining five-dimensional and single-dimensional search strategies, which represent the five arms of a starfish in high- and low-dimensional problems, respectively. The exploitation phase employs a two-directional parallel search strategy to reflect the hunting behavior of starfish. In addition, the regeneration mechanism imitates the generation of new individuals by taking the weakest individual in the population as a reference. This mechanism helps the algorithm move closer to the global optimum and provides a strategy for escaping local minima. SFOA has demonstrated competitive performance in both low-dimensional and high-dimensional problems, as well as in real-world engineering design problems.

### 2.2. Fitness–Distance Balance

Fitness–Distance Balance (FDB) is a selection method for metaheuristic algorithms developed by Kahraman et al. in 2020 to enhance search performance [20]. It plays a significant role in improving the performance of Metaheuristic Search (MHS) algorithms by guiding the search process and facilitating the selection of informative solution candidates within population-based structures. Selection strategies used in MHS algorithms are generally classified into three categories: greedy, probabilistic (e.g., roulette wheel and tournament selection), and random. Each method affects the balance between exploration and exploitation differently, thereby influencing the algorithm’s convergence behavior.

In the greedy selection method, solutions in the population are ranked according to their fitness value, which is important for selection. In the probabilistic selection method, an individual’s probability of being selected is determined by its fitness value using mechanisms such as roulette wheel or tournament selection. In the random selection method, individuals are selected randomly from the population, unlike the other methods where selection is based on fitness-related criteria.

The FDB method operates in a manner similar to greedy selection strategies. What sets it apart from conventional greedy methods is that it does not focus solely on the fitness value when making selections. Instead, the selected individual is determined using a score function that jointly considers the fitness component and the distance to the best solution $X_{\mathrm{best}}$ in the population. Thus, FDB considers not only high-quality solution candidates but also candidates located in different regions of the search space. This reduces the likelihood of becoming trapped in local optima and improves the exploration–exploitation balance. To calculate the FDB score of each solution candidate in the population, the following steps are performed.

(a) The objective value of each solution candidate is calculated. In a population of size *m*, each candidate consists of *n* design variables. The *i*-th solution candidate can be expressed as
  (1) $$X_{i}=\left[x_{1}^{\left(i\right)},x_{2}^{\left(i\right)},x_{3}^{\left(i\right)},\ldots ,x_{n}^{\left(i\right)}\right].$$
  The objective value of each solution candidate is calculated using the objective function f(·). As a result of this process, the vector containing the objective values of all solution candidates in the population is obtained as follows:
  (2) $$P\equiv [x_{11}\;\cdots \;x_{1n};\;\vdots \;\ddots \;\vdots ;\;x_{m1}\;\cdots \;x_{mn}],$$
  (3) $$FV=\left[f\left(X_{1}\right),f\left(X_{2}\right),f\left(X_{3}\right),\ldots ,f\left(X_{m}\right)\right].$$
  Here:
  - $f(X_{i})$ denotes the raw objective value corresponding to the *i*-th solution candidate,
  - FV is the vector containing the objective values of all individuals,
  - this vector serves as a fundamental input for calculating the FDB scores.
(b) The Euclidean distance of each solution candidate from the best individual in the current population, $X_{\mathrm{best}}$, is calculated. The aim of this step is to consider not only the candidates with good objective values but also those strategically positioned in different regions of the search space. Accordingly, the Euclidean distance between the *i*-th solution candidate $X_{i}$ and the best solution $X_{\mathrm{best}}$ can be expressed as
  (4) $$D\left(X_{i},X_{\mathrm{best}}\right)=\sqrt{\sum_{k=1}^{n}{\left(x_{k}^{\left(i\right)}-x_{k}^{\left(\mathrm{best}\right)}\right)}^{2}}.$$
  Here:
  - $D_{i}$ denotes the distance of the individual $X_{i}$ from $X_{\mathrm{best}}$,
  - $x_{k}^{\left(i\right)}$ represents the *k*-th design variable of the *i*-th individual,
  - $x_{k}^{\left(\mathrm{best}\right)}$ represents the corresponding variable of the best individual,
  - *n* is the number of decision variables.
  This distance value is the second component of the FDB score function. It is intended to enhance diversity and support the algorithm’s exploration capability. The use of Euclidean distance in this subsection follows the general FDB formulation adopted for the fixed FDBSFOA variant. The distance metric used in the dynamic dFDBSFOA variant is clarified separately in the dFDB scheduling subsection.
(c) The distance vector $D_{P}$, which is obtained by calculating the Euclidean distances of all solution candidates from the best individual $X_{\mathrm{best}}$, is defined as follows:
  (5) $$D_{P}={\left[\begin{array}{c} d_{1}\\ d_{2}\\ \vdots \\ d_{m} \end{array}\right]}_{m\times 1}={\left[d_{1},d_{2},\ldots ,d_{m}\right]}^{⊤}.$$
  Here:
  - $d_{i}=D\left(X_{i},X_{\mathrm{best}}\right)$ denotes the distance of the *i*-th solution candidate from the best individual,
  - *m* is the total number of solution candidates in the population,
  - $D_{P}\in R^{m\times 1}$ serves as the second fundamental component in the calculation of the FDB score function.
(d) The score function is calculated for each solution candidate using the objective-value vector in Equation (3) and the distance vector in Equation (5). Since all CEC benchmark functions and engineering design problems considered in this study are formulated as minimization problems, the raw objective values are transformed into maximization-oriented fitness scores before computing the FDB/dFDB score. Following the original FDB formulation, the normalized objective value is first calculated as
  (6) $${\mathrm{normG}}_{i}=\frac{f_{i}-f_{\min}}{f_{\max}-f_{\min}+\varepsilon },$$
  where $f_{i}$ is the raw objective value of the *i*-th candidate solution, and $f_{\min}$ and $f_{\max}$ are the minimum and maximum objective values in the current population, respectively. Here, ε denotes a small positive numerical constant introduced to prevent division by zero in degenerate cases where all candidate solutions have identical objective values, i.e., $f_{\max}=f_{\min}$. The corresponding maximization-oriented fitness component is then defined as
  (7) $${\mathrm{normFit}}_{i}=1-{\mathrm{normG}}_{i}.$$
  Therefore, lower objective values produce higher normalized fitness scores. The normalized distance component is computed as
  (8) $${\mathrm{normDist}}_{i}=\frac{d_{i}-d_{\min}}{d_{\max}-d_{\min}+\varepsilon },$$
  where $d_{i}$ denotes the distance of the *i*-th candidate from the current best solution, and $d_{\min}$ and $d_{\max}$ are the minimum and maximum distance values in the current population. Accordingly, the FDB score is calculated as
  (9) $$S_{i}=w\;{\mathrm{normFit}}_{i}+\left(1-w\right)\;{\mathrm{normDist}}_{i},\;i=1,2,\ldots ,m.$$
  Here:
  - $S_{i}$ represents the FDB score of the *i*-th solution candidate,
  - ${\mathrm{normFit}}_{i}$ is the minimization-oriented normalized fitness component,
  - ${\mathrm{normDist}}_{i}$ is the normalized distance component,
  - w∈[0,1] is the coefficient balancing the fitness and distance components,
  - *m* is the population size.
  This definition ensures that candidates with better minimization-oriented fitness values and informative spatial diversity relative to the current best solution receive higher FDB scores. This enables a balance between exploration and exploitation.
  The formation of the score vector is carried out after the calculation of the FDB scores for each solution candidate. The scores are then consolidated into a vector structure, thereby preparing them for the subsequent selection process. The vector containing the scores of all individuals, $S_{P}$, is defined as
  (10) $$S_{P}={\left[\begin{array}{c} s_{1}\\ s_{2}\\ \vdots \\ s_{m} \end{array}\right]}_{m\times 1}={\left[s_{1},s_{2},\ldots ,s_{m}\right]}^{⊤}.$$
  Here:
  - $S_{i}$ represents the FDB score of the *i*-th solution candidate, as defined in Equation (9),
  - $S_{P}\in R^{m\times 1}$ is the ordered score vector used in the selection process.
  This score vector reflects the relative quality and diversity of individuals within a population. It is used to select reference individuals to guide the search process.

### 2.3. Dynamic Fitness–Distance Balance

The Dynamic Fitness–Distance Balance (dFDB) selection method, proposed by Kahraman et al. in 2021 [21], enhances the performance of metaheuristic algorithms by modifying their selection mechanism. The dFDB method improves both exploration and convergence by dynamically adjusting the weighting coefficient employed in the original FDB method. In this approach, the score of each candidate solution is calculated by dynamically weighting the normalized fitness and distance components. The normalized fitness component reflects the objective quality of the candidate solution and therefore contributes mainly to exploitation, whereas the normalized distance component serves as a diversity-related indicator by measuring the spatial separation of the candidate from the current best solution. Consequently, as the search process unfolds, the selected individuals adaptively revise their guiding roles in accordance with the evolving demands of exploration and exploitation. The score value in the original dFDB method is computed as

(11) $$S_{i}=w_{\mathrm{dFDB}}\left(h\right)\;{\mathrm{normF}}_{i}+\left[1-w_{\mathrm{dFDB}}\left(h\right)\right]{\mathrm{normD}}_{i}.$$

Here:

- ${\mathrm{normF}}_{i}$ denotes the normalized fitness value of the *i*-th candidate solution,
- ${\mathrm{normD}}_{i}$ denotes the normalized distance of the *i*-th candidate solution from the best solution in the population,
- $w_{\mathrm{dFDB}}$ denotes the dynamic weighting coefficient.

The dynamic weighting coefficient in the original dFDB formulation is defined as

(12) $$w_{\mathrm{dFDB}}=\left(\frac{h}{\mathrm{maxFE}}\right)\left(1-lb\right)+lb.$$

Here, lb represents the lower bound of the $w_{\mathrm{dFDB}}$ coefficient. This constraint ensures that the fitness control does not fall below a predefined lower threshold during the search process. Thus, the randomness of the search process is minimized. The steps used to generate the adjustable-frequency $w_{\mathrm{dFDB}}$ coefficient are presented in the pseudocode in Algorithm 1.
**Algorithm 1** Pseudo-code to generate the frequency-adjustable $w_{\mathrm{dFDB}}$ signal  1: **Begin**  2: Assign the values of maxFE and *f*.  3: **if** (maxFE/f)≥1 **then**  4:       mod_ref←round(maxFE/f)  5:       **for** i=1 to maxFE **do**  6:             h←imodmod_ref  7:             $w_{\mathrm{dFDB}}\leftarrow \frac{h}{maxFE}\;\cdot \;\left(1-lb\right)+lb$  8:       **end for**  9: **end if**10: **End**

Using the pseudocode given in Algorithm 1, two *w* signals with frequencies of 1 and 10 are produced. These signals are then used to obtain frequency-adjusted $w_{\mathrm{dFDB}}$ values, after which the scores of the solution candidates are calculated using the original dFDB method as

(13) $$\begin{array}{cc} S_{P_{i}} & =w_{\mathrm{dFDB}}\;{\mathrm{normF}}_{i}\\ \; & \;+\left(1-w_{\mathrm{dFDB}}\right){\mathrm{normD}}_{P_{i}},\;i=1,\ldots ,m. \end{array}$$

The equations above summarize the original dFDB notation. In the proposed dFDBSFOA implementation, however, the dynamic coefficient is rewritten using α(t) and β(t) to explicitly distinguish the fitness and distance/diversity weights and to avoid ambiguity in the interpretation of *w*. Specifically, α(t) always represents the fitness weight, whereas β(t)=1−α(t) represents the distance/diversity weight. Thus, the dFDB score is consistently computed as

(14) $$S_{i}\left(t\right)=\alpha \left(t\right)\;{\mathrm{normFit}}_{i}+\left[1-\alpha (t)\right]{\mathrm{normDist}}_{i}.$$

In this formulation, early iterations use a lower fitness weight to emphasize distance-based diversity and exploration, whereas later iterations increase the fitness weight to strengthen exploitation near promising regions. This means that the search process is diversity-dominant during the early stages, reducing the risk of premature convergence, and becomes increasingly fitness-dominant in the later stages, allowing more precise convergence.

The fixed and scheduled fitness–distance weight coefficients can be summarized as follows: In the fixed FDB variant, the balance coefficient is set to α=0.5, matching the original FDB formulation; unlike the time-varying α(t) used in the dynamic dFDB formulation, this coefficient remains constant throughout the search process. This fixed, author-selected balanced setting assigns equal emphasis to the minimization-oriented fitness component and the distance/diversity component across different benchmark categories and engineering design problems. Therefore, the fixed FDB score is computed as

(15) $$S_{i}=0.5\;{\mathrm{normFit}}_{i}+0.5\;{\mathrm{normDist}}_{i}.$$

To further examine the influence of the dynamic scheduling range, an additional parameter sensitivity study is conducted while all other settings remain unchanged. In the dynamic formulation, α(t) denotes the fitness weight and β(t)=1−α(t) denotes the distance/diversity weight. Since the implementation updates the distance/diversity weight periodically, the CFG configurations are reported in terms of $\beta_{\max}$ and $\beta_{\min}$. Three configurations are considered: CFG1 (fitness-dominant, low diversity), CFG2 (balanced and close to the main experimental line), and CFG3 (diversity-preserving). The detailed definitions of these configurations are summarized in Table 3, and the corresponding sensitivity results are reported in the Results section.

#### Dynamic Fitness–Distance Scheduling in dFDB

In dFDBSFOA, the fitness–distance balance is not controlled by a manually tuned constant; instead, it is scheduled dynamically as a function of the search progress and updated periodically. Let *t* denote the current iteration and Gen denote the maximum number of iterations, or the iteration-equivalent form of the MaxFEs budget. The dFDB mechanism updates the scheduling coefficient every frequency steps by defining

(16) $$f_{x}=\mathrm{round}\left(\frac{Gen}{\mathrm{frequency}}\right),\;y=\mathrm{mod}\left(t,f_{x}\right).$$

In the implementation, the scheduled coefficient β(t) represents the distance/diversity weight. It is computed as a linearly decreasing function within the predefined interval $\left[\beta_{\min},\beta_{\max}\right]$:

(17) $$\beta \left(t\right)=\beta_{\max}-\left(\frac{y}{f_{x}}\right)\left(\beta_{\max}-\beta_{\min}\right),\;\beta \left(t\right)\in \left[\beta_{\min},\beta_{\max}\right].$$

The corresponding fitness weight is then obtained as

(18) α(t)=1−β(t).

Thus, early iterations assign a higher weight to the distance/diversity term to encourage exploration, whereas later iterations increasingly emphasize the fitness term to strengthen exploitation. This formulation removes the ambiguity between the fitness and distance weights: α(t) always denotes the fitness weight, and β(t) always denotes the distance/diversity weight.

Distance metric used in FDBSFOA and dFDBSFOA. The distance component in FDB/dFDB is used to quantify the spatial diversity of each candidate solution relative to the current best solution. In the general FDB framework, different distance metrics such as Euclidean, Manhattan, or Minkowski distance can be used depending on the structure of the target optimizer and the intended guide-selection behavior. In this study, the fixed FDBSFOA variant uses the Euclidean distance, which is consistent with the general FDB formulation:

(19) $$d_{i}^{\mathrm{Euc}}=\sqrt{\sum_{j=1}^{D}{\left(x_{i,j}-x_{\mathrm{best},j}\right)}^{2}}.$$

For the dynamic dFDBSFOA variant, the distance component is computed using the Manhattan, or absolute deviation, distance:

(20) $$d_{i}^{\mathrm{Man}}=\sum_{j=1}^{D}\left|x_{i,j}-x_{\mathrm{best},j}\right|.$$

This choice was made because the dFDB mechanism dynamically changes the relative contribution of fitness and distance during the search process. The Manhattan distance provides a simple component-wise additive measure of separation from the current best solution and is computationally suitable for repeated dynamic guide selection. Therefore, the two metrics are not used to define different optimization objectives; rather, they are used as diversity indicators within the same fitness–distance-aware selection principle. A systematic investigation of alternative distance metrics is beyond the scope of this study and is considered a promising direction for future work.

Accordingly, in the dFDBSFOA implementation, the Manhattan distance in Equation (20) is used as the distance component in the dynamic score calculation. Both the minimization-oriented fitness values and distances are normalized into [0,1] to ensure scale compatibility. The final dFDB score is then computed as

(21) $$s_{i}\left(t\right)=\alpha \left(t\right)\;{\hat{f}}_{i}+\beta \left(t\right)\;{\hat{d}}_{i},$$

where ${\hat{f}}_{i}$ denotes the normalized minimization-oriented fitness component and ${\hat{d}}_{i}$ denotes the normalized distance component. Equivalently, using β(t)=1−α(t), Equation (21) can be written as

(22) $$s_{i}\left(t\right)=\alpha \left(t\right)\;{\hat{f}}_{i}+\left[1-\alpha (t)\right]{\hat{d}}_{i}.$$

The candidate with the maximum score is selected or prioritized depending on the insertion point described in Table 4. This promotes candidates that are simultaneously promising in terms of objective quality and informative in terms of spatial diversity. If all fitness values become identical, that is, min(f)=max(f), a random index is selected to avoid degenerate selection pressure.

Finally, boundary violations are corrected using *random reinitialization within bounds*; that is, any variable exceeding its lower or upper limit is reassigned by uniform sampling in $\left[lb_{j},ub_{j}\right]$. This handling is applied uniformly throughout all experiments to preserve feasibility and ensure comparability across methods.

### 2.4. Development of SFOA with FDB and dFDB Mechanisms

This section presents the proposed enhancement of the SFOA algorithm by embedding the Fitness–Distance Balance (FDB) and Dynamic Fitness–Distance Balance (dFDB) mechanisms into its search lifecycle.

**Figure 2:**
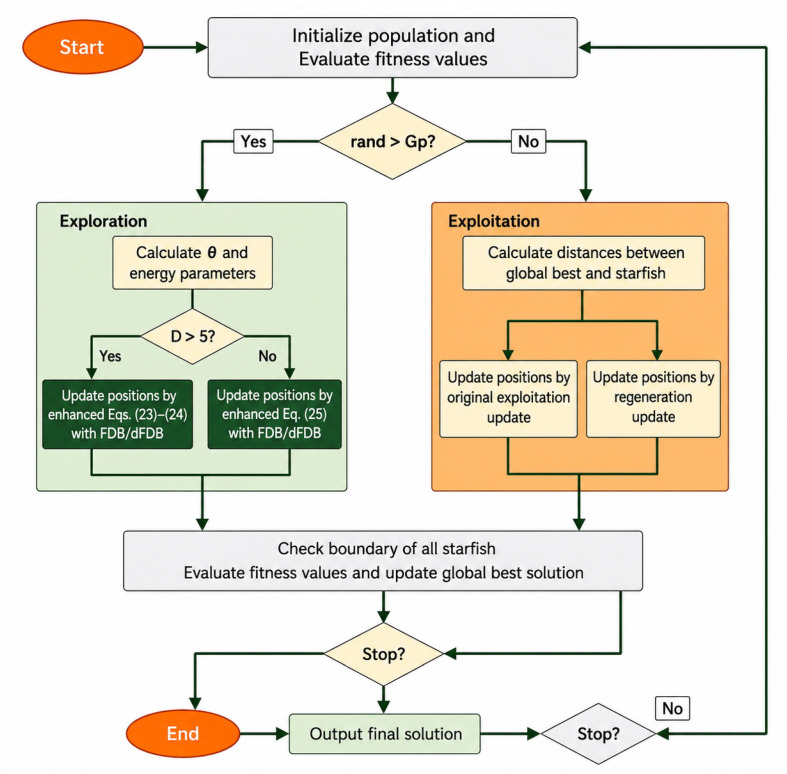
Flow diagram of the SFOA algorithm with FDB integration. The colors indicate the main SFOA search phases, the FDB-based guide-selection module, and the boundary-control stage.

The motivation for this integration is to improve the exploration–exploitation balance by enabling the algorithm to select informative solution candidates based on both fitness (exploitation) and spatial diversity (exploration). To this end, the mathematical structure of the original SFOA update process was analyzed, and the stages at which a fitness–distance-driven selection mechanism could be naturally inserted without altering the core dynamics of SFOA were identified. The overall workflow of the proposed SFOA framework with FDB-based guide selection is illustrated in Figure 2.

The analysis of the SFOA model (see Equations (23)–(25)) reveals multiple candidate points at which solution candidates are generated and/or selected during regeneration and movement phases. Table 4 summarizes these integration points and presents the corresponding equations in SFOA together with the FDB/dFDB-based candidate selection alternatives. Based on this structural template, multiple FDB/dFDB-augmented SFOA variants can be defined by activating different combinations of the identified selection points. In this study, a controlled set of variants is constructed to systematically assess how each insertion point and its combination affect convergence behavior, robustness, and computational overhead. Based on these update equations and the FDB/dFDB-based guide-selection mechanism summarized in Table 4, the pseudocode of the proposed SFOA variant is presented in Algorithm 2.
**Algorithm 2** Pseudo-code of the SFOA algorithm with FDB/dFDB integration**Require:** Algorithmic parameters *N*, $T_{\max}$, and $G_{p}$ **Ensure:** The global solution  1: Initialize input parameters, problem information, boundary values, etc.  2: Initialize positions by Equation (1) and evaluate the fitness values by Equation (3).  3: T←1  4: **while** 
$T<T_{\max}$ 
**do**  5:       **if** $\mathrm{rand}>G_{p}$ **then**                         ▷ Exploration phase  6:            Calculate θ by Equation (6) and energy $E_{b}$ by Equation (9).  7:            **for** all starfish *i* **do**  8:                  **if** D>5 **then**                   ▷ When the dimension is larger than 5  9:                       Generate five randomly selected *p*-dimensions among *D*. 10:                       **for** j←1 to 5 **do**            ▷ Update five randomly selected *p*-dimensions 11:                             Calculate $a_{1}$ by Equation (5). 12:                             Select the guide solution $Xpos_{\mathrm{FDB}/\mathrm{dFDB}}$ according to Table 4. $$\begin{array}{cc} \;\mathrm{newX}(i,jp1(j))= & {\mathrm{Xpos}}_{\mathrm{FDB}/\mathrm{dFDB}}\left(i,jp1\left(j\right)\right)\\ & -pm\left(\mathrm{bestSolution}\left(jp1\left(j\right)\right)-{\mathrm{Xpos}}_{\mathrm{FDB}/\mathrm{dFDB}}\left(i,jp1\left(j\right)\right)\right)\cos\left(\theta \right) \end{array}$$                                      ▷ Equation (23)$$\begin{array}{cc} \;\mathrm{newX}(i,jp1(j))= & {\mathrm{Xpos}}_{\mathrm{FDB}/\mathrm{dFDB}}\left(i,jp1\left(j\right)\right)\\ & -pm\left(\mathrm{bestSolution}\left(jp1\left(j\right)\right)-{\mathrm{Xpos}}_{\mathrm{FDB}/\mathrm{dFDB}}\left(i,jp1\left(j\right)\right)\right)\sin\left(\theta \right) \end{array}$$                                      ▷ Equation (24) 13:                             For the detailed integration point, see Table 4. 14:                       **end for** 15:                       Check the boundary of position by Equation (7). 16:                  **else**                   ▷ When the dimension is not larger than 5 17:                       Generate two random numbers $A_{1}$ and $A_{2}$. 18:                       Select a random dimension *p*. 19:                       Select the guide solution $Xpos_{\mathrm{FDB}/\mathrm{dFDB}}$ according to Table 4. $$\begin{array}{ccc} \;\mathrm{newX}(i,jp2)= & tEO\;\cdot \;{\mathrm{Xpos}}_{\mathrm{FDB}/\mathrm{dFDB}}\left(i,jp2\right) & \\ & +{\mathrm{rand}}_{1}\left(\mathrm{Xpos}\left(im\left(1\right),jp2\right)-{\mathrm{Xpos}}_{\mathrm{FDB}/\mathrm{dFDB}}\left(i,jp2\right)\right) & \;▷\mathrm{Equation}\;(25)\\ & +{\mathrm{rand}}_{2}\left(\mathrm{Xpos}\left(im\left(2\right),jp2\right)-{\mathrm{Xpos}}_{\mathrm{FDB}/\mathrm{dFDB}}\left(i,jp2\right)\right) & \end{array}$$ 20:                       For the detailed integration point, see Table 4. 21:                       Check the boundary of starfish by Equation (7). 22:                  **end if** 23:            **end for** 24:       **else**                              ▷ Exploitation phase 25:            Generate distances between the best position and starfish. 26:            **for** all starfish *i* **do** 27:                  Generate random number $r_{k}$, and update the position using Equation (11). 28:                  **if** i=N **then** 29:                       Update the position using Equation (12).            ▷ Regeneration phase 30:                  **end if** 31:                  Check the boundary of starfish by Equation (13). 32:            **end for** 33:       **end if** 34:       Calculate the fitness values for all starfish. 35:       Obtain the global best solution at iteration *T*. 36:       T←T+1 37: **end while** 38: Obtain the global solution.

To avoid the potential bias that may arise from ad hoc “best-case” selection and to ensure a principle-based design, the final configurations reported as FDBSFOA and dFDBSFOA were selected through a multi-criteria selection protocol rather than through isolated outcomes. Specifically, the candidate variants were evaluated under the same experimental budget and run protocol, that is, 21 independent runs, and the selection was guided by three criteria: overall effectiveness, robustness, and computational cost.

Effectiveness was assessed through Friedman ranking across benchmark functions. Robustness was evaluated by the dispersion across runs, for example, through standard deviation and interquartile range. Computational cost was assessed by the runtime overhead under identical maximum function evaluation budgets (MaxFEs). This protocol yields configurations that provide consistent performance improvements and stability while preserving cost-effectiveness. Furthermore, it enables a transparent interpretation of the contribution of each embedded mechanism.

Unless otherwise indicated, the fixed FDB formulation uses α=0.5, which assigns equal emphasis to the minimization-oriented fitness component and the distance/diversity component during candidate selection. In the dynamic dFDB formulation, the balance is controlled by the time-varying coefficients α(t) and β(t)=1−α(t), where α(t) denotes the fitness weight and β(t) denotes the distance/diversity weight. The sensitivity of these weighting coefficients is further examined in the Results section through a dedicated parameter sensitivity analysis, thereby ensuring that the reported performance is not attributable to an arbitrarily tuned setting.

(23) $$\mathrm{newX}\left(i,jp1\left(j\right)\right)=\mathrm{Xpos}\left(i,jp1\left(j\right)\right)-pm\left(\mathrm{bestSolution}(jp1(j))-\mathrm{Xpos}(i,jp1(j))\right)\cos\theta$$

(24) $$\mathrm{newX}\left(i,jp1\left(j\right)\right)=\mathrm{Xpos}\left(i,jp1\left(j\right)\right)-pm\left(\mathrm{bestSolution}(jp1(j))-\mathrm{Xpos}(i,jp1(j))\right)\sin\theta$$

(25) $$\begin{array}{cc} \mathrm{newX}(i,jp2)= & tEO\;\mathrm{Xpos}\left(i,jp2\right)+\mathrm{rand}1\left(\mathrm{Xpos}(im(1),jp2)-\mathrm{Xpos}(i,jp2)\right)\\ & +\mathrm{rand}2\left(\mathrm{Xpos}(im(2),jp2)-\mathrm{Xpos}(i,jp2)\right) \end{array}$$

### 2.5. Design Rationale and Variant Selection Protocol

A structured design study was conducted to integrate FDB and dFDB mechanisms into SFOA, thereby avoiding ad hoc trial-and-error design. As illustrated in Table 4, the twelve variants under consideration represent controlled combinations of three design axes. The first is distance-aware selection pressure, which aims to preserve diversity and mitigate premature convergence. The second is fitness-driven exploitation pressure, which aims to intensify local refinement near promising regions. The third is the dynamic scheduling mechanism of dFDB, which adjusts the relative influence of fitness and distance during the search in accordance with the changing requirements of exploration and exploitation.

To select the final configurations, namely FDBSFOA and dFDBSFOA, used in the benchmark and engineering evaluations, a three-criterion selection protocol was employed. The first criterion was overall effectiveness, assessed by Friedman ranking across functions. The second criterion was robustness, quantified by the standard deviation (STD) and/or the interquartile range (IQR) across 21 independent runs. The third criterion was computational cost, measured as the runtime overhead relative to the baseline SFOA under the same MaxFEs budget. This protocol ensures that the final structures are selected based on consistent multi-criteria evidence rather than isolated best-case outcomes. It also provides an explicit rationale that links each component to the intended search behavior.

### 2.6. Algorithm Complexity

Computational complexity is a significant criterion for evaluating the practical applicability of metaheuristic optimizers. In addition to solution quality, it is necessary to examine whether an algorithmic enhancement introduces excessive computational overhead or remains cost-effective. Therefore, this section provides both a theoretical complexity analysis and an empirical normalized runtime-complexity assessment for the baseline SFOA and its FDB- and dFDB-integrated variants.

The theoretical analysis clarifies the additional cost introduced by the fitness–distance-based guide-selection mechanism, whereas the empirical timing analysis quantifies the implementation-level overhead using the normalized complexity measure.

#### 2.6.1. Theoretical Complexity of FDB/dFDB-Based Guide Selection

Let *N* denote the population size, *D* the problem dimension, MaxFEs the maximum number of function evaluations, $C_{f}$ the cost of one objective-function evaluation, *K* the number of active FDB/dFDB insertion points in a given variant, and *I* the number of guide-selection recalculation steps during the search process. This interpretation is also consistent with recent FDB-based metaheuristic improvement studies. In such designs, the FDB module generally performs four operations: identifying the current best solution, computing the distance between each candidate and the best solution, normalizing the fitness and distance vectors, and selecting the candidate with the highest fitness–distance score. Since the distance computation dominates these operations, a single FDB call requires O(ND) vector operations. Therefore, integrating FDB/dFDB increases the per-iteration vector-processing load but does not change the dominant asymptotic complexity class of the underlying optimizer. In the present study, this explains why the proposed FDBSFOA and dFDBSFOA variants may require additional runtime in some cases, while remaining computationally practical under the same MaxFEs-based evaluation budget.

In the baseline SFOA, candidate positions are updated according to the original SFOA search equations. Ignoring the problem-dependent objective-function evaluation cost, the vector-level update operations scale linearly with the dimension. Therefore, the baseline computational cost can be expressed as

$$O_{\mathrm{SFOA}}=O\left(MaxFEs\;\cdot \;C_{f}+MaxFEs\;\cdot \;D\right).$$

In the proposed FDBSFOA and dFDBSFOA variants, the additional cost originates from the FDB/dFDB guide-selection mechanism. Each FDB/dFDB call consists of four main operations: identifying the current best candidate, calculating the distance between each population member and the current best solution, normalizing the fitness and distance vectors, and selecting the candidate with the highest fitness–distance score. The distance calculation over *N* candidates in a *D*-dimensional search space requires O(ND) operations, while normalization and score calculation are linear in *N*. Thus, the complexity of one FDB/dFDB guide-selection call is

$$O_{\mathrm{FDB}-\mathrm{call}}=O\left(ND\right).$$

If the FDB/dFDB mechanism is activated at *K* insertion points and recalculated *I* times during the optimization process, the additional complexity becomes

$$O_{\mathrm{extra}}=O\left(KIND\right).$$

Accordingly, the total complexity of the proposed FDB/dFDB-enhanced variants can be written as

$$O_{\mathrm{FDB}/\mathrm{dFDB}-\mathrm{SFOA}}=O\left(MaxFEs\;\cdot \;C_{f}+MaxFEs\;\cdot \;D+KIND\right).$$

Under the same MaxFEs-based stopping criterion, the FDB/dFDB mechanism increases the number of vector-level operations per iteration but does not change the dominant asymptotic order of the optimizer. The dynamic coefficient update in dFDB is a scalar operation and is negligible compared with the O(ND) distance-score calculation. Therefore, the main overhead of both FDBSFOA and dFDBSFOA is caused by repeated fitness–distance score calculation and guide selection.

#### 2.6.2. Empirical Normalized Complexity Measurement

For a fair and implementation-aware comparison, three timing components were measured. $T_{0}$ represents the runtime of a fixed reference program and serves as a hardware-dependent baseline. $T_{1}$ represents the runtime of repeated objective-function evaluations, and $T_{2}$ represents the total runtime of the complete optimizer under the same evaluation setting. The normalized complexity is computed as

$$\mathrm{Complexity}=\frac{\bar{T_{2}}-T_{1}}{T_{0}},$$

where $\bar{T_{2}}$ is the average runtime over five independent executions. This metric reduces hardware dependence and highlights the algorithmic overhead beyond objective-function evaluation cost.

All timing experiments were performed in MATLAB R2023a on a workstation equipped with a 13th Gen Intel i9-13900 CPU and 48 GB RAM. The complexity measurements are conducted on representative CEC2022 benchmark functions F1, F7, and F12 for D∈{10,20}, using identical evaluation budgets across algorithms. Each method was executed five times to obtain $\bar{T_{2}}$.

Discussion.

Table 5, Table 6 and Table 7 show that embedding FDB/dFDB into SFOA introduces a controlled runtime overhead relative to the baseline under identical evaluation budgets. This behavior is consistent with the theoretical analysis: each FDB/dFDB guide-selection call requires an additional O(ND) distance-score calculation, but the overall asymptotic order of the optimizer remains unchanged under the same MaxFEs-based stopping criterion.

Across CEC2022 F1, F7, and F12 for both D=10 and D=20, the enhanced variants generally exhibit higher normalized complexity than the baseline SFOA, as expected. However, the increase remains within a moderate range and does not indicate a prohibitive computational burden. The dFDB variants include an additional dynamic weighting mechanism, but this update is scalar and negligible compared with the population-level distance computation.

Table 8 reports the first high-dimensional normalized complexity results on CEC2017 F1 for D=30, D=50, and D=100. Unlike the CEC2022 low-dimensional cases, the absolute normalized complexity values are substantially larger because both the evaluation budget and the dimensionality increase with *D*. Nevertheless, the results indicate that the FDB/dFDB-integrated variants remain within the same order of magnitude as the baseline SFOA across all dimensions. Table 9 reports the normalized runtime complexity results on CEC2017 F15 for D=50 and D=100. For D=50, several FDB/dFDB variants show lower normalized complexity than the baseline SFOA. This behavior can be attributed to implementation-level runtime variability, MATLAB execution behavior, and stochastic search trajectories, and does not contradict the theoretical analysis. For D=100, most FDB/dFDB variants introduce additional runtime relative to SFOA, which is expected because the fitness–distance-based guide-selection mechanism requires extra O(ND) vector operations. Nevertheless, most dFDB variants except dFDB_110 remain close to the baseline order of magnitude, supporting the interpretation that the proposed mechanisms introduce a controlled and interpretable computational overhead rather than a prohibitive computational burden.

For D=30, several FDB/dFDB variants show slightly lower normalized complexity than the baseline SFOA. This does not contradict the theoretical analysis, because normalized runtime measurements are affected by implementation-level factors such as MATLAB execution variability, memory behavior, vectorization efficiency, and stochastic search trajectories. Theoretically, each FDB/dFDB call introduces an additional O(ND) distance-score calculation, but this additional vector-processing cost does not change the dominant asymptotic order of the optimizer under the same MaxFEs-based stopping criterion.

For D=50 and D=100, most enhanced variants show a moderate increase relative to SFOA, which is expected because the distance-score calculation becomes more expensive as the dimension increases. However, the increase remains controlled for most configurations and supports the claim that FDB/dFDB guide selection adds a practical computational overhead rather than a prohibitive complexity burden. To further examine whether the additional distance-score calculation causes a dimension-dependent runtime burden, Table 8, Table 9 and Table 10 extend the normalized complexity analysis to representative high-dimensional CEC2017 functions. These functions represent unimodal/basic, multimodal, and hybrid landscapes, respectively, and provide a direct assessment of scalability under increased dimensionality.

The high-dimensional results in Table 8, Table 9 and Table 10 show that the proposed FDB/dFDB variants remain computationally practical as *D* increases. Although several enhanced variants exhibit higher normalized complexity than the baseline SFOA, the results remain within the same order of magnitude, supporting the theoretical claim that FDB/dFDB increases vector-processing cost but does not change the dominant asymptotic complexity class.

Table 10 presents the normalized complexity results on CEC2017 F7 for D=30, D=50, and D=100. The results show that the FDB/dFDB-based variants generally introduce an additional runtime cost compared with the baseline SFOA, which is expected because the guide-selection mechanism requires extra fitness–distance score calculations. However, the increase remains within the same order of magnitude for all dimensions. In particular, several dFDB variants, such as dFDB_171, dFDB_179, dFDB_183, dFDB_205, and dFDB_209, show relatively stable normalized complexity values across D=30 and D=50, while the D=100 case naturally produces larger values due to the increased evaluation budget and dimensionality.

The additional vector-processing cost should be interpreted together with the performance improvements reported in the benchmark and engineering-design experiments. The FDB/dFDB mechanisms replace purely random or fitness-only guidance with a more informative guide-selection strategy that jointly considers solution quality and spatial diversity. Therefore, the proposed variants provide a favorable trade-off between computational cost and optimization effectiveness, particularly in terms of exploration–exploitation balance, robustness, and convergence behavior.

## 3. Experimental Settings

The following configurations were adopted to ensure a fair and reproducible evaluation of the considered algorithms. The optimization process was terminated according to the maximum number of objective function evaluations (MaxFEs). For the CEC benchmark suites, the evaluation budget was defined as MaxFEs = 10,000 × *D*, where *D* denotes the problem dimension. Each test problem was assessed through 21 independent runs using distinct random seeds, and the resulting performance data were subjected to statistical analysis. For pairwise algorithm comparisons, the Wilcoxon signed-rank test was employed, whereas Friedman ranking was used for multiple-algorithm comparisons [22,23,24], with the significance level set to α=0.05.

To examine scalability across problem sizes, the CEC benchmark suites were evaluated at multiple dimensions: CEC2017 at D=10,30,50, and 100 [42], CEC2020 at D=30,50, and 100 [43], and CEC2022 at D=10 and 20 [44]. The IEEE CEC2017, CEC2020, and CEC2022 benchmark suites were used to assess the effectiveness of the proposed algorithms under diverse problem characteristics [45]. These suites encompass four main categories of continuous optimization problems: unimodal, multimodal/basic, hybrid, and composition. The general structures and compositions of the benchmark suites used in this study are summarized in Table 11, Table 12 and Table 13.

For the constrained engineering design problems, the evaluation budget was also set to MaxFEs= 10,000 × D, and the same experimental protocol of 21 independent runs was maintained. For all benchmarks and engineering problems, the search was initialized uniformly within the prescribed lower and upper bounds of each decision variable. These bounds were taken directly from the benchmark or problem definitions and therefore define the feasible search region. During the search, boundary violations were handled uniformly across all compared algorithms using random reinitialization; that is, any component exceeding its lower or upper bound was reassigned to a uniformly sampled value within the corresponding interval. This strategy prevents invalid solutions from biasing the search while preserving comparability among algorithms.

All benchmark experiments were executed using the official CEC function implementations, and the reported performance was computed using the conventional error definition $f\left(x\right)-f^{*}$, that is, with the bias removed, consistent with the CEC reporting protocol. All experiments were conducted in MATLAB R2023a on a system equipped with a 13th Gen Intel Core i9-13900 processor running at 2.00 GHz.

Benchmark verification (sanity checks).

For functions where near-zero errors or very rapid convergence were observed, additional sanity checks were performed to exclude implementation artifacts. Specifically, we (i) verified objective values at randomly sampled decision vectors against the outputs of the official CEC code, (ii) confirmed that the reported error strictly followed the bias-removed formulation $f\left(x\right)-f^{*}$, and (iii) ensured that the observed behavior persisted across the 21 independent seeds, thereby ruling out deterministic initialization or seeding artifacts. These checks support the conclusion that the reported outcomes reflect genuine algorithmic convergence under the specified MaxFEs budget rather than coding or seeding issues.

### Benchmark Test Suites Used in Experimental Studies

To evaluate the performance of metaheuristic-based search algorithms comprehensively, it is necessary to test them on diverse problem types and across search spaces of varying dimensionality, including low-, medium-, and high-dimensional cases. In the literature, continuous-valued optimization problems are typically categorized into four main groups: unimodal, multimodal, hybrid, and composition problems [42,46]. Unimodal problems have relatively simple search spaces without local optimum traps and are therefore primarily used to evaluate the exploitation capability of algorithms. In contrast, multimodal problems contain many local optima and are suitable for assessing exploration ability [47]. Hybrid and composition problems are more complex and are widely used to evaluate an algorithm’s ability to balance exploration and exploitation. Composition problems, in particular, provide a challenging environment for assessing whether an algorithm can reach the global optimum without being trapped in local solutions.

In this study, a broad experimental framework was established using the CEC2017, CEC2020, and CEC2022 suites to evaluate the proposed methods across different levels of difficulty and dimensionality. The overall structure of each suite, including the distribution of functions across the four categories, is provided in Table 11, Table 12 and Table 13, which summarize the benchmark characteristics used throughout the experimental analyses.

## 4. Results and Discussion

This section presents the findings of the experimental analysis of the developed algorithms. The analyses are organized under two main themes. The first evaluates the impact of the proposed Fitness–Distance Balance (FDB) and enhanced dFDB strategies on the SFOA algorithm. In this context, the original SFOA algorithm and its variants integrated with the FDB and dFDB approaches were tested comparatively. These evaluations provide a detailed analysis of algorithmic performance across different problem types and dimensions. The results suggest that the dFDB mechanism more effectively guides the search process and improves solution quality, thereby identifying the most efficient configuration for the SFOA algorithm.

It should be noted that several CEC functions include known shifts and biases with predefined global optimum values. Therefore, when an algorithm approaches the known optimum within numerical tolerance, the reported error may become extremely small or may appear as zero after rounding or formatting. All results were obtained from 21 independent runs with randomized initialization, and the reported statistics were computed directly from raw run-wise outputs without manual post-processing.

### 4.1. Optimizing the Performance of the Starfish Algorithm via Dynamic Fitness–Distance Balance

Within the scope of this study, twelve algorithmic variants were developed based on the standard SFOA algorithm by integrating FDB and dFDB mechanisms. This subsection presents comparative performance analyses of the standard SFOA algorithm and its FDB-based version, as well as the twelve variants derived using the dFDB strategy, including four FDB-based and eight dFDB-based structures.

In this study, the SFOA variant integrated with the FDB mechanism is referred to as *FDB_Case*, whereas algorithms that incorporate the dFDB strategy with different structures are denoted as *dFDB_Case*. Section 2 provides detailed information regarding the structural configurations and updated formulations of these variants.

This section is divided into three main subsections. The first presents statistical performance analyses of all algorithms and identifies the most successful variant based on significant differences. The second provides a detailed examination of convergence behavior. The third analyzes and compares the computational complexity of each variant. The results for CEC2022, CEC2020, and CEC2017 are summarized in Table 14, Table 15, and Table 16, respectively.

In addition to the Wilcoxon pairwise comparisons, the Friedman average-ranking results of the fixed FDBSFOA and dynamic dFDBSFOA variants are reported in Table 17 and Table 18, respectively.

The performance of the proposed SFOA–FDB variants was compared with that of the original SFOA on the CEC2022 test functions. The results were statistically analyzed using the Wilcoxon signed-rank test at a significance level of p<0.05. The test functions were grouped into four main categories: unimodal (F1), multimodal (F2–F5), hybrid (F6–F8), and composition (F9–F12). For the unimodal function F1, FDB integration provided a limited yet consistent improvement in convergence speed. Among the FDB variants, SFOA_FDB_Case_1_205 achieved the lowest error values. For the multimodal functions (F2–F5), the FDB variants were notably superior, particularly in higher-dimensional scenarios. Specifically, SFOA_FDB_Case_1_136 consistently yielded the best results for F3, whereas SFOA_FDB_Case_1_205 was the top performer for F4. For the hybrid functions (F6–F8), which involve complex search spaces and diverse subproblem structures, the adaptive exploration–exploitation balance of the FDB variants was advantageous, with notable improvements observed particularly for F6 and F8. In the composition functions (F9–F12), which represent the most challenging test group, SFOA_FDB_Case_1_183 and SFOA_FDB_Case_1_205 exhibited statistically significant superiority over the original SFOA in higher-dimensional settings. Overall, the FDB mechanism improved performance over a broad range of problems, from simple unimodal functions to complex composition functions, and exhibited particularly notable superiority in higher-dimensional multimodal, hybrid, and composition functions. The corresponding Wilcoxon pairwise comparison results are presented in Table 14.

**Figure 3:**
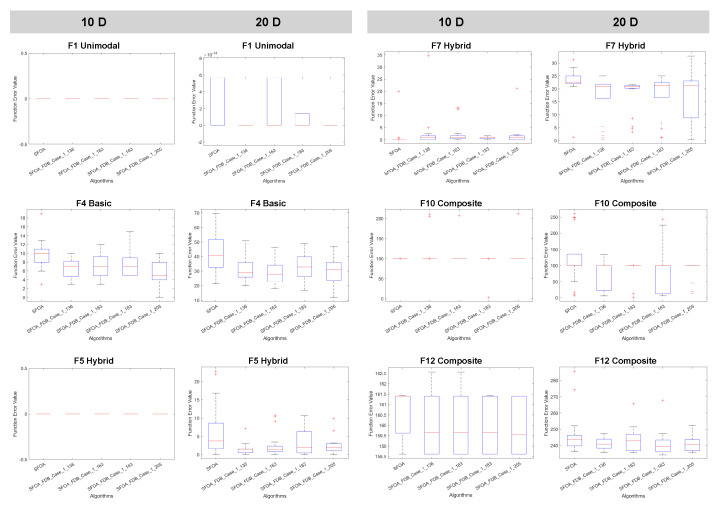
CEC2022 FDBSFOA boxplots for unimodal, basic, hybrid, and composition functions.

**Figure 4:**
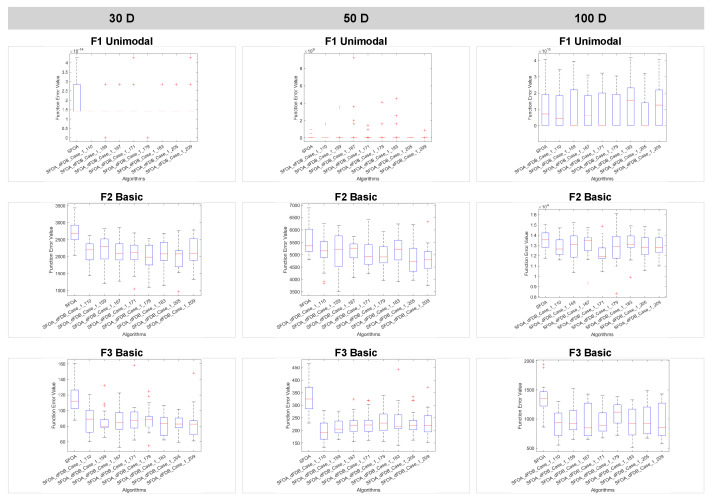
CEC2020 dFDBSFOA boxplots for unimodal and basic functions.

**Figure 5:**
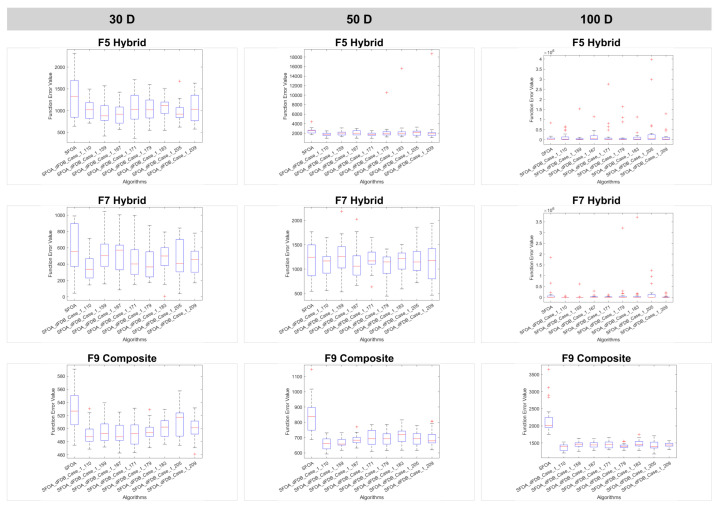
CEC2020 dFDBSFOA boxplots for hybrid and composition functions.

**Figure 6:**
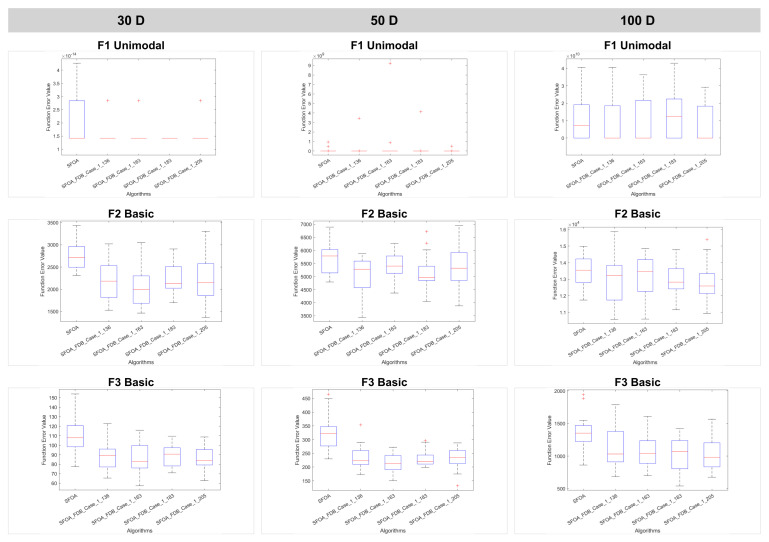
CEC2020 FDBSFOA boxplots for unimodal and basic functions.

**Figure 7:**
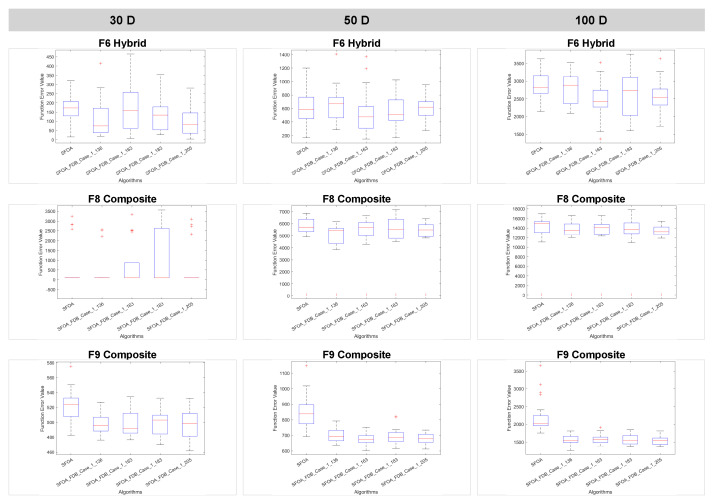
CEC2020 FDBSFOA boxplots for hybrid and composition functions.

**Figure 8:**
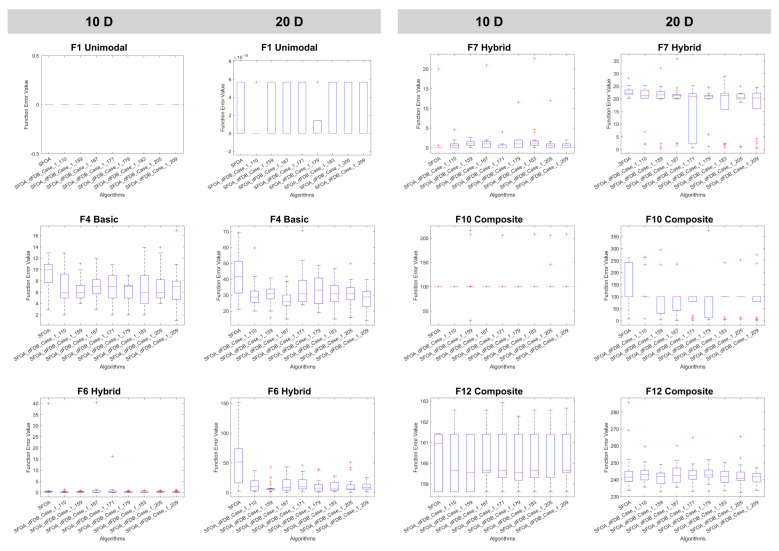
CEC2022 dFDBSFOA boxplots for unimodal, basic, hybrid, and composition functions.

Table 19 reports the post-hoc comparison results of the FDB/dFDB-based SFOA variants against the baseline SFOA on CEC2017. The baseline SFOA is included only to report its average rank; pairwise *p*-values and adjusted *p*-values are not applicable for SFOA because it is the control algorithm and is not compared against itself. The baseline SFOA obtains an average rank of 11.8966, whereas all FDB/dFDB-based variants obtain lower average ranks. Moreover, all Holm-adjusted comparisons remain statistically significant at α=0.05. These results indicate that the proposed FDB/dFDB-based guide-selection mechanisms provide statistically significant improvements over the baseline SFOA on the CEC2017 benchmark suite after post-hoc correction. The performance of the dFDB-based SFOA variants was also evaluated on the CEC2022 test functions in 10- and 20-dimensional settings. In the 20-dimensional setting, all variants demonstrated statistically significant superiority over the original SFOA. In particular, SFOA_dFDB_Case_1_171 and SFOA_dFDB_Case_1_205 performed especially well on unimodal (F1), multimodal (F3, F4, and F5), and hybrid (F7 and F8) problems, achieving statistically better results on six functions (see Figure 3, Figure 4, Figure 5, Figure 6, Figure 7 and Figure 8).

The remaining variants also exhibited superiority on F1, F3, F4, F7, and F8. However, in the 10-dimensional setting, the number of problems showing improvement decreased notably. The improvements were concentrated mainly on the multimodal F4 and the composition F11 functions. The hybrid F7 problem was the only function on which all variants were at a disadvantage. Overall, the dFDB strategy provided substantial performance improvements, particularly for higher-dimensional problems, whereas its influence was more limited in lower-dimensional settings.

On the CEC2020 test suite, the dFDB-based variants were notably superior to the baseline SFOA, especially in the 30D and 50D settings. In the 30D setting, the most successful variant, Case_1_110, achieved wins on the F1 unimodal, F2–F3 basic, F5–F7 hybrid, and F8–F9 composition functions. Overall, the improvements were concentrated mainly in the basic and composition classes, with F10 remaining the most challenging function. In the 50D setting, no losses were observed among the variants, and the strongest performances were obtained by Case_1_179 and Case_1_209. The gains were distributed across the basic (F2–F3), hybrid (F5–F6), and composition (F8–F9) functions (see Figure 4, Figure 5, Figure 6 and Figure 7). In the 100D setting, all variants operated without losses; however, the victories became more selective. In particular, F3 from the basic class and F9 from the composition class were improved by almost all variants. In contrast, the unimodal F1 was improved only by Case_1_205, whereas the hybrid F7 was improved only by Case_1_110. Across all dimensions, the dFDB mechanism provided broad superiority in medium-dimensional problems while maintaining stable and targeted improvements in higher-dimensional scenarios. No variant exhibited a statistically significant performance decline. Table 15 presents the Wilcoxon pairwise comparison results between the standard SFOA and its variants on the CEC2020 test suite.

The evaluations on the CEC2020 test suite further indicate that the FDB-based SFOA variants provide statistically significant improvements over the baseline SFOA across all dimensional settings (30D, 50D, and 100D). In the 30D scenario, SFOA_FDB_Case_1_136 achieved the highest number of wins and performed best on the unimodal (F1), basic (F2 and F3), hybrid (F7), and composition (F9) functions, whereas the remaining variants exhibited comparable superiority, especially on F1, F2, F3, and F9, indicating consistent gains in the basic and composition classes. In the 50D scenario, SFOA_FDB_Case_1_136 again emerged as the strongest variant, outperforming its competitors on the unimodal (F1), basic (F2 and F3), and composition (F8 and F9) functions. The other variants mostly prevailed on F1, F3, and F9, suggesting that the FDB mechanism remained effective for both basic and complex landscapes as dimensionality increased. In the 100D scenario, the number of wins decreased overall; nevertheless, SFOA_FDB_Case_1_205 attained the best results, showing superiority on the unimodal (F1), basic (F2 and F3), and composition (F8 and F9) functions, whereas SFOA_FDB_Case_1_163 and SFOA_FDB_Case_1_183 remained competitive, particularly on the basic F3 and composition F9 functions (see Figure 4, Figure 5, Figure 6 and Figure 7).

Overall, the FDB-based variants demonstrated improved performance across a broader range of problems in the 30D and 50D settings. In contrast, in the 100D setting, the gains were concentrated mainly in the basic and composition functions. This suggests that the FDB mechanism makes strong and consistent contributions, especially in medium-dimensional optimization problems, while still providing selective yet stable improvements in higher-dimensional problems.

Taken together, the Friedman rankings and significance analyses of the CEC2022 test results show that the FDB and dFDB variants generally outperform the original SFOA algorithm. Notably, in 20-dimensional problems, all FDB and dFDB configurations achieved lower average ranking values than SFOA, and most of these differences were statistically significant (p<0.05). Within the dFDB group for 20D problems, the top three algorithms were SFOA_dFDB_Case_1_209, SFOA_dFDB_Case_1_205, and SFOA_dFDB_Case_1_179. Within the FDB group, the leading variants were SFOA_FDB_Case_1_183, SFOA_FDB_Case_1_163, and SFOA_FDB_Case_1_136. For the 10D problems, SFOA_dFDB_Case_1_167 achieved the best rank among the dFDB variants, whereas SFOA_FDB_Case_1_205 exhibited performance comparable to that of the original SFOA (see Figure 3 and Figure 8). This improvement trend, which becomes stronger as dimensionality increases, indicates that the proposed FDB and dFDB strategies are more effective in high-dimensional search spaces and therefore contribute significantly to the scalability of metaheuristic optimization.

The results of the Wilcoxon pairwise comparisons on the CEC2017 benchmark problems indicate that the FDB- and dFDB-based variants significantly outperform the baseline SFOA algorithm. While the baseline SFOA did not achieve statistically significant superiority on any problem, the FDB- and dFDB-based variants demonstrated superiority on 13 to 19 out of 29 problems. The baseline SFOA outperformed the variants only in a few cases, such as F4, F12, and F25. When evaluated according to dimensionality, the improvements in the 10D setting were limited, with most results remaining comparable. From the 30D problems onward, improvements became more pronounced, particularly for the FDB-based variants such as SFOA_FDB_Case_1_136, which achieved superiority without losses. For the 50D problems, the dFDB-based variants were especially notable, with algorithms such as SFOA_dFDB_Case_1_179 and SFOA_dFDB_Case_1_205 outperforming the baseline on almost all functions. The corresponding Wilcoxon pairwise comparison results are presented in Table 16.

In the 100D problems, the dFDB-based variants generally maintained their superiority. When the results are examined by problem class, the baseline SFOA produced average outcomes for unimodal functions. The FDB variants made limited contributions, whereas the dFDB-based variants exhibited stronger convergence behavior. For multimodal functions, all variants showed improvement, particularly the dFDB-based algorithms such as SFOA_dFDB_Case_1_159 and SFOA_dFDB_Case_1_167, which yielded stable results. The dominance of dFDB in hybrid functions was particularly notable. For example, SFOA_dFDB_Case_1_171 and SFOA_dFDB_Case_1_179 demonstrated clear superiority across many 50D scenarios. In composition functions, the FDB-based variants produced strong results, but the dFDB variants achieved the highest success overall. In summary, the FDB mechanism provided consistent contributions in low- and medium-dimensional problems, whereas the dFDB mechanism substantially improved the optimization performance of SFOA, especially on high-dimensional, multimodal, hybrid, and composition functions.

A joint evaluation of the CEC2017, CEC2020, and CEC2022 benchmark problems reveals that both the FDB and dFDB strategies outperform the baseline SFOA algorithm across multiple dimensions and problem types. The CEC2017 results show that, although the differences are relatively limited in low-dimensional cases, the FDB variants consistently outperform the baseline algorithm, especially on problems with 30 dimensions or more. Meanwhile, the dFDB variants dominate the baseline on nearly all functions at 50D and 100D. The results obtained from the CEC2020 and CEC2022 benchmarks further confirm this improvement, as the Friedman scores and significance values indicate that both strategies improve average performance and provide consistent and repeatable results across different problem classes. In particular, for 100-dimensional problems, the FDB and dFDB variants exhibit a more balanced exploration–exploitation behavior than the original SFOA. The steeper slopes observed in the convergence curves further show that the enhanced variants approach the optimum more rapidly. Overall, the three benchmark suites consistently demonstrate that the FDB-based variants improve performance mainly in low- and medium-dimensional problems, whereas the dFDB-based variants are stronger and more diverse in high-dimensional, multimodal, hybrid, and composition functions. Together, these findings indicate that the proposed variants substantially enhance the optimization performance of SFOA across a broad range of dimensions and problem categories (see Table 17 and  Table 18).

### 4.2. Convergence Analysis

**Figure 9:**
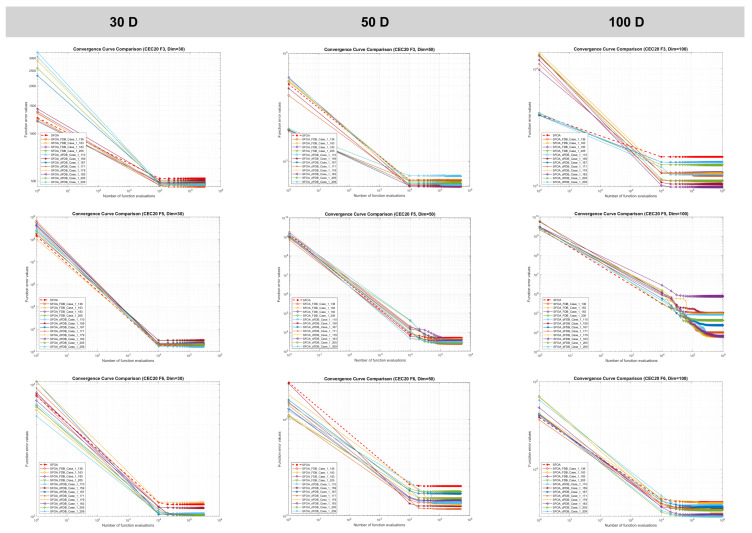
CEC2020 convergence charts (Part 1).

**Figure 10:**
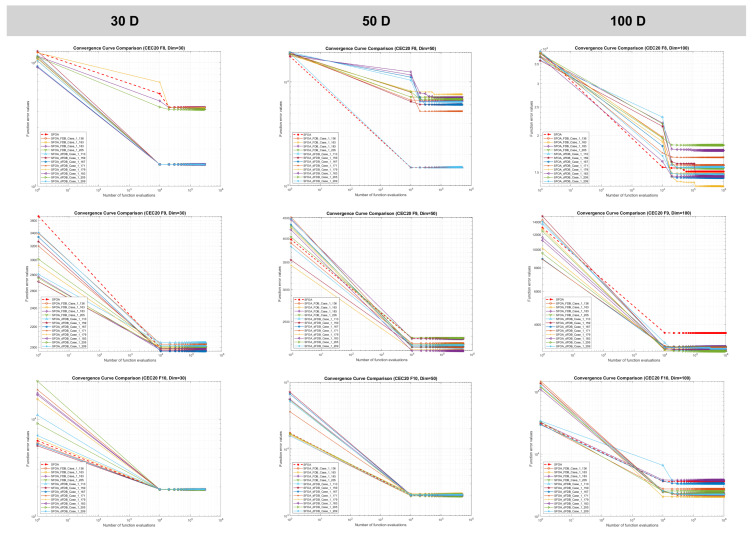
CEC2020 convergence charts (Part 2).

This subsection presents the convergence performance of the baseline SFOA algorithm and its thirteen variants developed using the FDB and dFDB methods. To illustrate their search performance, four different problem types were selected from the CEC2020 benchmark suite. Convergence curves were plotted for the F1 unimodal, F2–F4 basic, F5–F7 hybrid, and F8–F10 composition groups based on function error values obtained at 30, 50, and 100 dimensions. The convergence behavior of these selected CEC2020 problems is depicted in Figure 9 and Figure 10.

For the unimodal F1 problem, both the baseline SFOA and its thirteen variants converged to an error value below 10 in the 30D and 50D search spaces. In the 100D case, SFOA_dFDB_Case_1_209 demonstrated superior convergence performance by achieving a lower error value than the other variants. Most of the remaining variants also produced better convergence results than the original SFOA. Consequently, SFOA_dFDB_Case_1_209 demonstrated a more effective neighborhood search capability than its competitors.

For the F2 basic-type problem in the 30D search space, the baseline SFOA produced intermediate results. In contrast, SFOA_FDB_Case_1_136 achieved an error value close to zero and therefore delivered the best performance. In the 50D case, the baseline SFOA exhibited the highest convergence error among all compared methods, whereas all other variants showed better convergence behavior. Notably, SFOA_dFDB_Case_1_159 produced an error value close to zero and yielded the best outcome. In the 100D case, SFOA_dFDB_Case_1_183 demonstrated the most successful convergence performance.

For the basic-type function F3, the baseline SFOA exhibited the weakest convergence behavior in the 30D and 100D search spaces, while all other variants outperformed it. In contrast, in the 50D case, SFOA_dFDB_Case_1_159 achieved the best convergence performance among all tested variants. For the hybrid-type function F5, SFOA yielded the poorest convergence in the 50D scenario, whereas all other variants performed better. In the 100D scenario, multiple variants surpassed SFOA and attained the best convergence results, while in the 30D scenario SFOA_dFDB_Case_1_209 was the only variant that converged faster than SFOA. For the hybrid-type function F6, SFOA again demonstrated the weakest convergence in both the 50D and 100D settings. In the 30D setting, SFOA_dFDB_Case_1_179 performed worse than the original SFOA, whereas SFOA_dFDB_Case_1_171 delivered the best convergence performance in the 50D case. Overall, many variants converged more effectively than SFOA across the remaining dimensions. Finally, for the hybrid-type function F7, the convergence trends were largely similar across SFOA and its variants in the 30D and 50D scenarios, whereas in the 100D scenario the convergence speed of SFOA decreased and the variants achieved better performance.

In the 30D scenario for the F8 composition-type problem, SFOA lagged behind its competitors, while the other variants achieved better convergence results. In the 50D scenario, SFOA and SFOA_dFDB_Case_1_209 achieved the best convergence performance. In the 100D scenario, SFOA_dFDB_Case_1_179 obtained the best convergence results, whereas the baseline SFOA ranked sixth among all variants.

In the 50D and 100D scenarios, the baseline SFOA exhibited worse convergence performance than its competitors for the F9 composition-type problem. In the 30D scenario, it ranked eleventh. The best convergence results were achieved by SFOA_dFDB_Case_1_167, SFOA_dFDB_Case_1_183, and SFOA_FDB_Case_1_205 in the 30D, 50D, and 100D scenarios, respectively.

For the F10 composition-type problem, the baseline SFOA and its variants achieved similar convergence results in the 30D and 50D scenarios. However, in the 100D scenario, the convergence performance of SFOA declined, resulting in a thirteenth-place ranking. The SFOA_FDB_Case_1_163 variant achieved the best convergence result in the 100D scenario.

In summary, the convergence analysis on the CEC2020 test functions suggests that the FDB and dFDB variants offer substantial enhancements over the original SFOA algorithm across various problem types and dimensions. For unimodal and basic problems, such as F1–F3, especially in higher-dimensional search spaces (100D), variants such as SFOA_dFDB_Case_1_209, SFOA_dFDB_Case_1_183, and SFOA_dFDB_Case_1_159 achieved substantially lower error values than the original SFOA. Similarly, many variants on hybrid (F5–F7) and composition (F8–F10) problems exhibited better convergence trends in both early iterations and final solutions. These results indicate that the FDB and dFDB mechanisms improve the exploration–exploitation balance of SFOA and therefore enhance convergence speed and solution quality, particularly for high-dimensional and complex problems, while reducing the final error.

#### Convergence Behavior on Selected CEC2022 Functions

To further examine the search dynamics of the proposed variants, representative convergence curves are reported for selected CEC2022 functions. These curves complement the numerical comparison tables by illustrating how the function error values decrease as the number of function evaluations increases. In particular, CEC2022 F3, F4, F6, F7, and F10 were selected because they provide representative cases in which the proposed FDB/dFDB-based SFOA variants show better or competitive convergence behavior relative to the baseline SFOA. The curves were obtained for D=20 over 21 independent runs, using the same evaluation budget and experimental settings as in the main benchmark comparison. In addition to the baseline SFOA and the proposed FDB/dFDB-based SFOA variants, the convergence comparison also includes several external FDB/dFDB-based metaheuristic optimizers, including FDB_AEO, dFDB_MRFO, FDB_SFS, and dFDB_SFS. The FDB_SFS and dFDB_SFS variants are supported by previous FDB/dFDB-based stochastic fractal search studies [48,49], FDB_AEO is supported by the FDB-based artificial ecosystem optimization study [50], and dFDB-based guidance follows the dynamic FDB selection framework [21].

**Figure 11:**
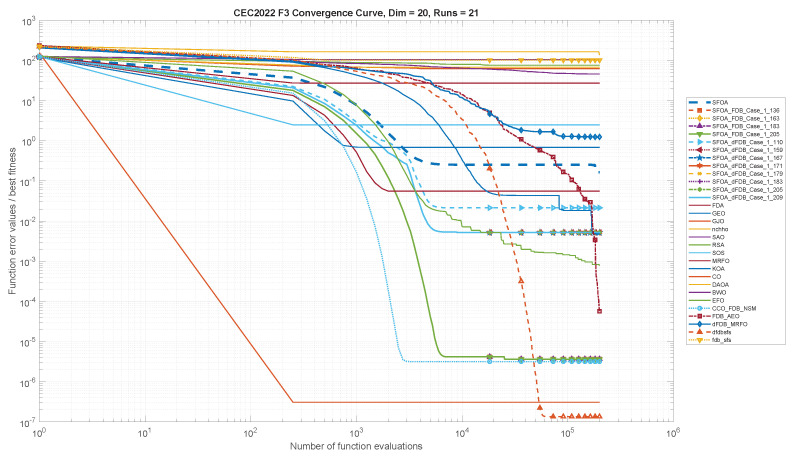
Convergence curve on CEC2022 F3 with D=20 over 21 independent runs.

**Figure 12:**
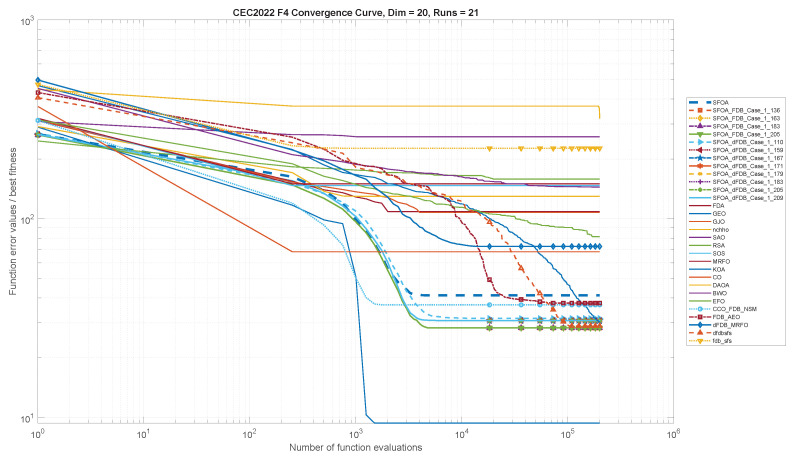
Convergence curve on CEC2022 F4 with D=20 over 21 independent runs.

**Figure 13:**
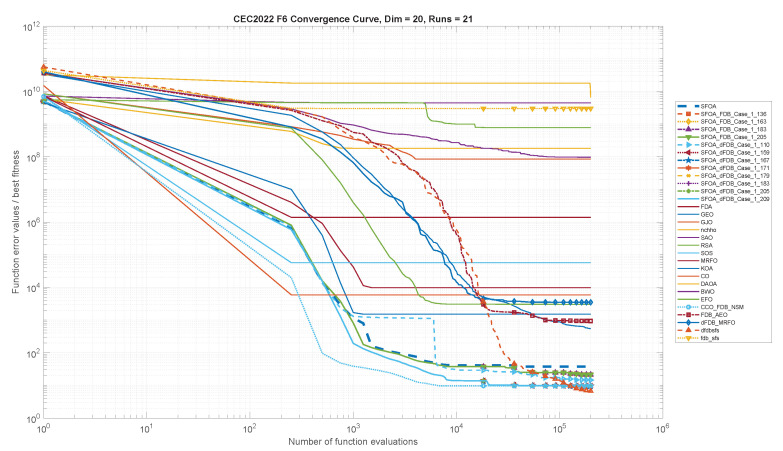
Convergence curve on CEC2022 F6 with D=20 over 21 independent runs.

**Figure 14:**
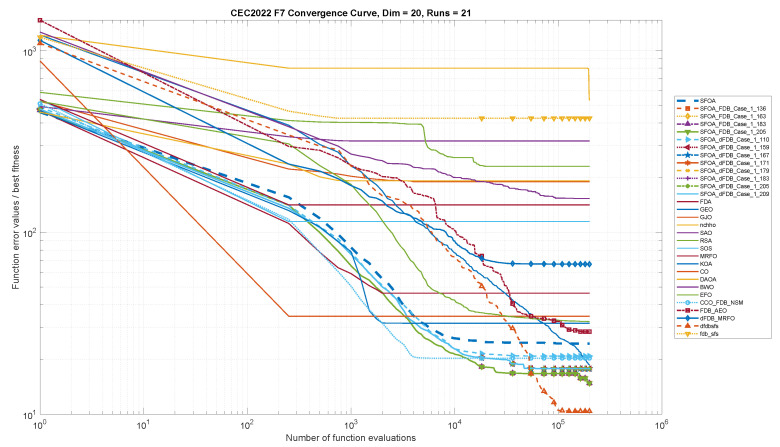
Convergence curve on CEC2022 F7 with D=20 over 21 independent runs.

**Figure 15:**
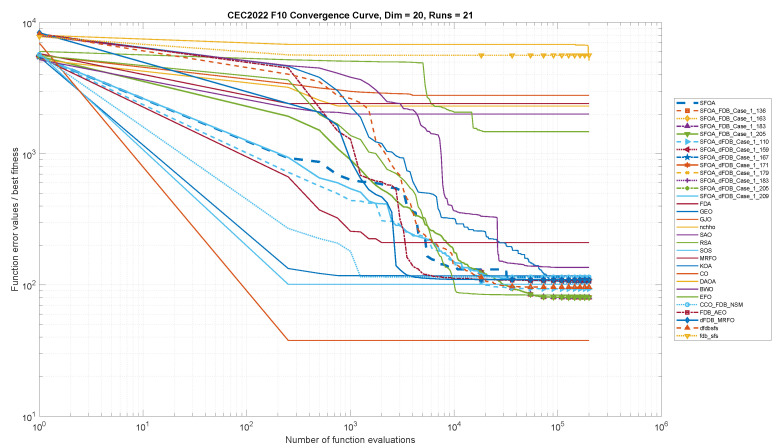
Convergence curve on CEC2022 F10 with D=20 over 21 independent runs.

As shown in Figure 11, Figure 12, Figure 13, Figure 14 and Figure 15, the proposed FDB/dFDB-based variants generally exhibit faster or more stable reductions in function error values than the baseline SFOA on the selected CEC2022 functions. The convergence behavior indicates that incorporating fitness–distance-based guide selection helps maintain a more effective exploration–exploitation balance during the search process. In particular, the selected curves demonstrate that the proposed mechanisms can improve not only the final error values but also the convergence trajectory across different types of benchmark landscapes. These visual observations are consistent with the numerical results and are further supported by the nonparametric statistical analyses.

After evaluating the proposed variants on benchmark suites, their applicability to constrained real-world optimization is further examined through engineering design problems.

### 4.3. Engineering Applications

All constrained engineering design problems considered in this study were handled using a static penalty-based constraint-handling strategy. The same constraint-handling procedure and penalty settings were applied consistently to all compared algorithms for each engineering design problem to ensure a fair comparison. The tension/compression spring problem includes four inequality constraints, the pressure vessel problem includes four inequality constraints, the cantilever beam problem includes one inequality constraint, the blending–pooling–separation problem includes 32 equality-type constraints, and the four-stage gear box problem includes 86 inequality constraints.

For all engineering design problems, the lower and upper bounds of the design variables were taken from the corresponding MATLAB implementation and applied identically to all compared algorithms. The problem-specific bounds are reported under each engineering problem description to improve reproducibility.

For inequality constraints, all constraints were expressed in the standard form $g_{j}\left(x\right)\leq 0$. For a candidate solution *x*, the unscaled total positive constraint violation is defined as

(26) $$CV_{\mathrm{ineq}}\left(x\right)=\sum_{j=1}^{m}\max\left(0,g_{j}\left(x\right)\right).$$

In the MATLAB implementation, the function get-penalty accumulates only positive constraint violations and multiplies the accumulated violation by $10^{6}$. Therefore, the penalty value returned by an inequality-constraint function is

(27) $$P_{\mathrm{ineq}}\left(x\right)=10^{6}CV_{\mathrm{ineq}}\left(x\right).$$

Since the returned penalty value is again multiplied by $10^{6}$ in the penalized objective function, the effective penalized objective used during optimization is

(28) $$\begin{array}{cc} F_{\mathrm{pen}}\left(x\right) & =f_{0}\left(x\right)+10^{6}P_{\mathrm{ineq}}\left(x\right)\\ \; & =f_{0}\left(x\right)+10^{12}CV_{\mathrm{ineq}}\left(x\right). \end{array}$$

where $f_{0}\left(x\right)$ denotes the original unpenalized objective function.

For equality-type constraints, which are used in the blending–pooling–separation problem, a tolerance of $10^{-6}$ was applied. Given equality constraints $h_{j}\left(x\right)=0$, the unscaled equality violation is defined as

(29) $$CV_{\mathrm{eq}}\left(x\right)=\sum_{j=1}^{m}\left\{\begin{array}{cc} |h_{j}\left(x\right)|, & |h_{j}\left(x\right)|>10^{-6},\\ 0, & |h_{j}\left(x\right)|\leq 10^{-6}. \end{array}\right.$$

In the MATLAB implementation, the function get_penalty_equals_zero applies this tolerance and multiplies the accumulated equality violation by $10^{6}$. Since the returned value is again multiplied by $10^{6}$ in the penalized objective, the effective penalized objective for the blending–pooling–separation problem is

(30) $$F_{\mathrm{pen}}\left(x\right)=f_{0}\left(x\right)+10^{12}CV_{\mathrm{eq}}\left(x\right).$$

A candidate solution is considered feasible if its corresponding unscaled constraint violation satisfies $CV\left(x\right)\leq 10^{-6}$. Although separate feasibility-rate and average-constraint-violation statistics were not stored as independent output variables in the original experimental logs, the reported engineering-design tables are based on the penalized objective $F_{\mathrm{pen}}\left(x\right)$. Consequently, any constraint violation is directly reflected in the reported Best, Worst, Mean, Median, Std, and IQR values. This is why some competing algorithms exhibit extremely large Worst, Mean, or IQR values in the engineering-design results: such values correspond to heavily penalized constraint-violating runs. In contrast, the proposed variants generally show lower objective values and smaller dispersion, indicating more stable behavior under the same penalty-based constraint-handling strategy. The constraint types, effective penalty formulations, variable bounds, and discrete-handling rules of the engineering design problems are summarized in Table 20.

Eleven popular metaheuristic algorithms were used for comparison. Specifically, the proposed SFOA-based methods were compared with BWO [51], KOA [52], DAOA [53], MRFO [2], SOS [54], FDA [55], GEO [56], GJO [57], SAO [58], and RSA [59].

#### 4.3.1. Cantilever Beam Design Problem

**Figure 16:**
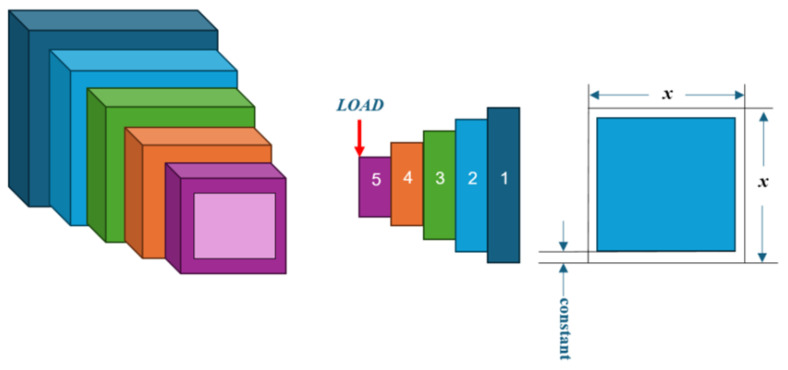
Cantilever beam design problem. The numbers indicate the five design variables used in the mathematical model.

The cantilever beam design problem is a widely studied real-world engineering optimization benchmark, as illustrated in Figure 16. The objective is to minimize the structure’s weight while ensuring that the vertical displacement at the free end remains within a certain range. This problem involves five design variables, $x=(x_{1},x_{2},x_{3},x_{4},x_{5})$ [60]. The mathematical formulation adopted in this study is expressed in Equation (32).

Bounds.

The design variables were bounded as follows:

(31) $$0.01\leq x_{i}\leq 100,\;i=1,2,\ldots ,5.$$

(32) $$\begin{array}{cc} \min\; & f\left(x\right)=0.0624\;\left(x_{1}+x_{2}+x_{3}+x_{4}+x_{5}\right),\\ s.t.\; & g_{1}\left(x\right)=\frac{61}{x_{1}^{3}}+\frac{37}{x_{2}^{3}}+\frac{19}{x_{3}^{3}}+\frac{7}{x_{4}^{3}}+\frac{1}{x_{5}^{3}}-1\leq 0,\\ \; & 0.01\leq x_{i}\leq 100,\;i=1,2,\ldots ,5. \end{array}$$

**Figure 17:**
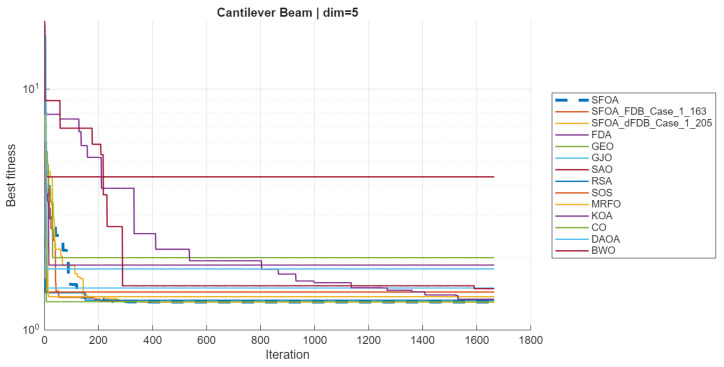
Convergence behavior of the compared algorithms on the Cantilever Beam design problem (dim = 5).

As reported in Table 21, SFOA and its enhanced variants (FDBSFOA and dFDBSFOA) achieve the best objective value of 3.679444444 with zero IQR and an almost zero standard deviation, indicating perfectly stable performance across runs. In contrast, most competitors yield larger medians and noticeably higher dispersion (IQR), reflecting weaker robustness under the penalty based constraint handling. Moreover, several algorithms (e.g., SAO, RSA, DAOA, and BWO) exhibit extremely large statistics, suggesting frequent convergence to heavily penalized infeasible solutions. The convergence profiles in Figure 17 further confirm that the proposed variants rapidly approach the best known region in early iterations and maintain steady refinement thereafter, demonstrating strong exploitation capability without sacrificing run to run reliability.

#### 4.3.2. Tension/Compression Spring Design

**Figure 18:**
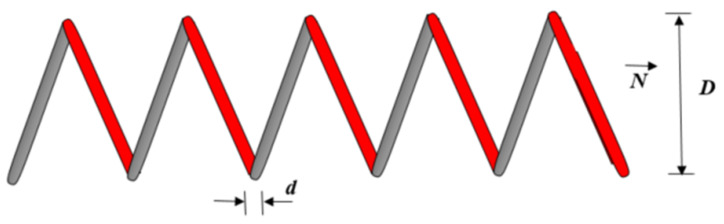
Tension/compression spring design problem.

As shown in Figure 18, the tension/compression spring design problem involves minimising the weight of the spring while adhering to constraints on shear stress, surge frequency and deflection. The problem involves three decision variables: the number of active coils ($P=x_{1}$), the mean coil diameter ($D=x_{2}$), and the wire diameter ($d=x_{3}$) [61]. The mathematical formulation of the problem is given in (34). **Bounds.** The design variables were bounded as follows in the MATLAB implementation:

(33) $$0.05\leq x_{1}\leq 2.0,\;0.25\leq x_{2}\leq 1.3,\;2.0\leq x_{3}\leq 15.0.$$

(34) $$\begin{array}{cc} \min_{x}\; & f\left(x\right)=x_{1}^{2}\;x_{2}\;\left(x_{3}+2\right),\\ s.t.\; & g_{1}\left(x\right)=1-\frac{x_{2}^{3}x_{3}}{71,785\;x_{1}^{4}}\leq 0,\\ \; & g_{2}\left(x\right)=\frac{4x_{2}^{2}-x_{1}x_{2}}{12,566(x_{2}x_{1}^{3}-x_{1}^{4})}+\frac{1}{5108x_{1}^{2}}-1\leq 0,\\ \; & g_{3}\left(x\right)=1-\frac{140.45\;x_{1}}{x_{2}^{2}x_{3}}\leq 0,\\ \; & g_{4}\left(x\right)=\frac{x_{1}+x_{2}}{1.5}-1\leq 0,\\ \; & 0.05\leq x_{1}\leq 2.00,\;0.23\leq x_{2}\leq 1.30,\;2.00\leq x_{3}\leq 15.0. \end{array}$$

**Figure 19:**
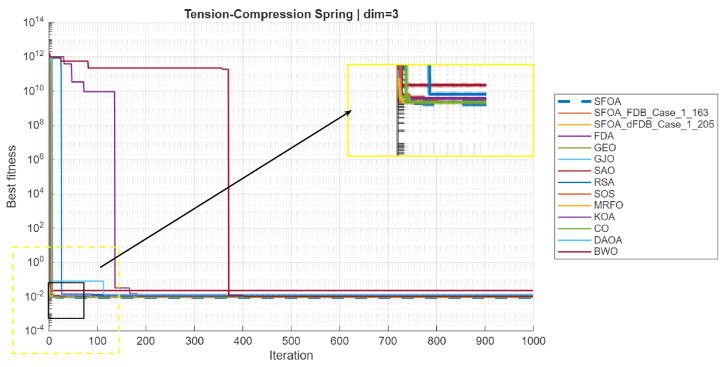
Convergence behavior of the compared algorithms on the Tension/Compression Spring design problem (dim = 3).

Table 22 shows that SFOA and its enhanced variants (FDBSFOA and dFDBSFOA) consistently reach the best objective value of 0.00890241. Notably, the dispersion measures confirm superior robustness: the proposed variants exhibit extremely small standard deviations and near zero IQR values, with FDBSFOA achieving the smallest IQR ($1.35\times 10^{-13}$), indicating the most stable run to run behavior. In contrast, competing algorithms generally yield higher medians and larger IQR values, reflecting weaker reliability under constraint handling. Furthermore, several methods (e.g., SAO and BWO) produce extraordinarily large worst/mean values, implying frequent convergence to heavily penalized infeasible solutions. The convergence curves in Figure 19 corroborate these findings, where the proposed variants rapidly attain the best region in early iterations and maintain steady refinement, whereas some competitors show stagnation or instability before approaching acceptable solutions.

#### 4.3.3. Pressure Vessel Design

**Figure 20:**
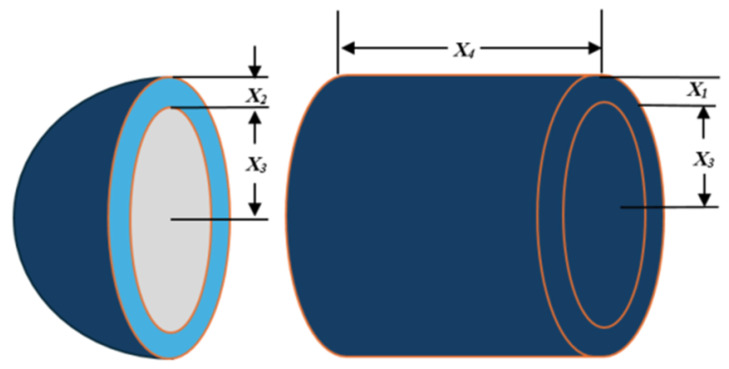
Cylindrical pressure vessel design problem.

The pressure vessel design problem is a well-known real-world engineering benchmark [62]. It involves four decision variables: shell thickness ($T_{s}=x_{1}$), head thickness ($T_{h}=x_{2}$), inner radius ($R=x_{3}$), and cylindrical length ($L=x_{4}$) (37). The main objective is to minimize the vessel’s total manufacturing cost while satisfying four nonlinear constraints related to material strength, fabrication feasibility and volume requirements. Figure 20 shows the design problem of a cylindrical pressure vessel.

**Bounds.** The design variables were bounded as follows:

(35) $$0.0625\leq x_{1}\leq 6.1875,\;0.0625\leq x_{2}\leq 6.1875,$$

(36) $$10\leq x_{3}\leq 200,\;10\leq x_{4}\leq 200.$$

**Implementation note.** For reproducibility, we clarify that the pressure vessel problem was evaluated according to the continuous bounded MATLAB implementation used in this study. Although the classical pressure vessel benchmark is often treated as a mixed discrete–continuous problem in which the thickness variables are multiples of 0.0625, no additional rounding operation was applied to $x_{1}$ and $x_{2}$ in the present code. The same encoding, bounds, objective function, four inequality constraints, and penalty settings were applied consistently to all compared algorithms.

(37) $$\begin{array}{cc} \min\;\; & f\left(x\right)=0.6224\;x_{1}x_{3}x_{4}+1.7781\;x_{2}x_{3}^{2}\\ \; & \;+3.1661\;x_{1}^{2}x_{4}+19.84\;x_{1}^{2}x_{3},\\ s.t.\;\; & g_{1}\left(x\right)=-x_{1}+0.0193\;x_{3}\leq 0,\\ \; & g_{2}\left(x\right)=-x_{2}+0.00954\;x_{3}\leq 0,\\ \; & g_{3}\left(x\right)=-\pi x_{3}^{2}x_{4}-\frac{4}{3}\pi x_{3}^{3}+1,296,000\leq 0,\\ \; & g_{4}\left(x\right)=x_{4}-240\leq 0,\\ \; & 0.0625\leq x_{1},x_{2}\leq 6.1875,\\ \; & 10\leq x_{3},x_{4}\leq 200. \end{array}$$

**Figure 21:**
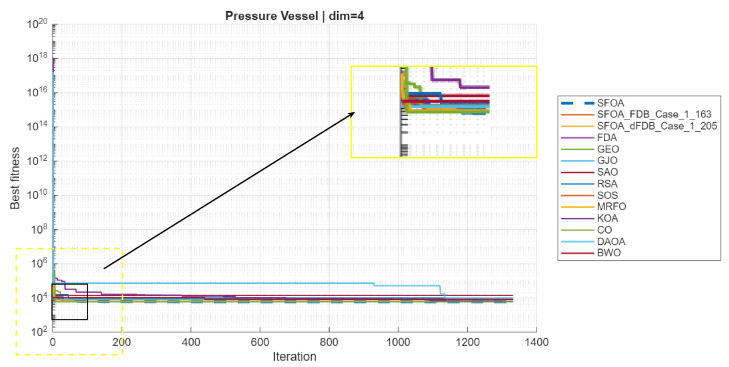
Convergence behavior of the compared algorithms on the Pressure Vessel design problem (dim = 4).

**Implementation note.** For reproducibility, we clarify that the pressure vessel problem was evaluated according to the continuous bounded MATLAB implementation used in this study. Although the classical pressure vessel benchmark is often treated as a mixed discrete–continuous problem in which the thickness variables are multiples of 0.0625, no additional rounding operation was applied to $x_{1}$ and $x_{2}$ in the present code. The same encoding, bounds, objective function, constraints, and penalty settings were applied consistently to all compared algorithms. As can be seen from Table 23, the proposed variants achieve the lowest objective values with markedly improved robustness. In particular, FDBSFOA and dFDBSFOA attain the best solution (around 5885.333) while exhibiting very small dispersion (Std and IQR), indicating highly consistent performance across runs. Compared with the baseline SFOA, the enhanced variants reduce variability substantially, suggesting that the FDB/dFDB mechanisms improve stability under penalty-based constraint handling. In contrast, the competing algorithms generally produce considerably larger medians and much higher IQR values (e.g., FDA, GEO, GJO, RSA, DAOA), reflecting weaker reliability and frequent convergence to inferior regions. The convergence curves in Figure 21 support these observations, showing that the proposed variants rapidly approach the best-known region and maintain stable refinement, whereas several competitors stagnate or converge more slowly to higher-cost solutions.

#### 4.3.4. Blending–Pooling–Separation Problem

The Blending–Pooling–Separation (BPS) problem models a process-systems design/operation task in which multiple streams are blended, intermediate pools are used for buffering/mixing, and downstream separation units deliver products that must satisfy quality and feasibility requirements [63]. The problem is challenging for metaheuristic optimizers because feasibility is governed by a coupled set of nonlinear process constraints, including flow balances, capacity limits, and operational/quality specifications, which typically lead to a narrow feasible region.

**Figure 22:**
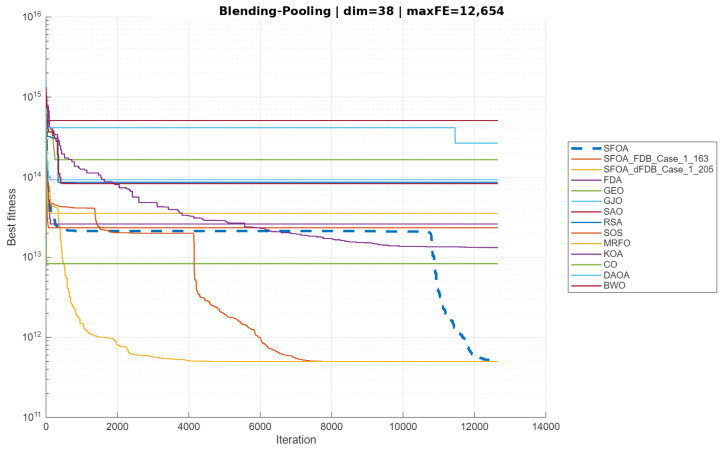
Convergence curves (log-scale) on the Blending-Pooling problem, D=38.

**Decision variables and evaluation.** A candidate solution is represented by a 38-dimensional decision vector $x=(x_{1},x_{2},\ldots ,x_{38})$. In the MATLAB implementation, all lower bounds are set to zero, and the upper bounds are defined in Equation (40). The objective is evaluated directly from selected components of x, specifically $x_{5}$ and $x_{13}$, and feasibility is enforced using an equality-constraint penalty term.
**Objective function.** Following the exact MATLAB implementation used in this study, the objective function to be minimized is
  (38) $$\min_{x}\;f\left(x\right)=0.9979+0.00432\;x_{5}+0.01517\;x_{13}+10^{6}\;\Phi \left(x\right),$$
**Problem size summary.** The BPS problem is evaluated using D=38 continuous decision variables and 32 equality-type constraints. These constraints are evaluated by Φ(x)=BlendingPooling(x) and handled using the equality-constraint penalty formulation in Equation (30). Since this routine internally applies the $10^{6}$ multiplier to the unscaled equality violation with a tolerance of $10^{-6}$, the effective penalized objective used during optimization is equivalent to $f_{0}\left(x\right)+10^{12}CV_{\mathrm{eq}}\left(x\right)$, where $CV_{\mathrm{eq}}\left(x\right)$ is defined in Equation (29).
**Bounds.** The blending–pooling–separation problem was implemented with D=38 decision variables. The lower bound vector was set to
  (39) $$lb=0_{1\times 38},$$
  and the upper bound vector was defined as
  (40) $$\begin{array}{cc} ub=[\; & 90,150,90,150,90,90,150,90,90,90,150,150,\\ \; & 90,90,150,90,150,90,150,90,1,1.2,1,1,1,\\ \; & 0.5,1,1,0.5,0.5,0.5,1.2,0.5,1.2,1.2,0.5,1.2,1.2]. \end{array}$$
**Constraint handling.** Feasibility is handled via the static equality-constraint penalty strategy described in Equation (30). For feasible solutions, Φ(x)=0, whereas for infeasible solutions, Φ(x)>0, and the penalized objective in Equation (38) increases according to the accumulated equality-constraint violation. The same bounds, objective function, equality-constraint tolerance, and penalty settings were applied consistently to all compared algorithms.
**Problem size summary.** The BPS problem is evaluated using D=38 continuous decision variables and 32 equality-type constraints. These constraints are aggregated in Φ(x)=BlendingPooling(x) and handled using the equality-constraint penalty formulation in Equation (30).
Results and Discussion. Table 24 reports the statistical outcomes on the Blending–Pooling problem with D=38 and 21 independent runs, while Figure 22 depicts the corresponding best so far convergence trends on a log scale. The convergence behavior shows a distinct separation: most competing optimizers stagnate early at high objective levels (typically $10^{13}$–$10^{15}$), whereas the SFOA family continues to reduce the penalized objective throughout the search budget, indicating more effective feasibility-driven exploitation under tight evaluations. In terms of best-achieved solution quality, the enhanced variants lead the comparison, where SFOA_dFDB_Case_1_205 attains the overall best value (4.999994214580522 × 10^11^), closely followed by SFOA_FDB_Case_1_163 (4.999994375026695 × 10^11^) and the baseline SFOA (5.101887305803711 × 10^11^). Robustness is also improved by the proposed mechanisms: SFOA_FDB_Case_1_163 yields the lowest median (3.870369405115016 × 10^12^), and both enhanced variants substantially reduce dispersion compared with SFOA (IQR: 2.409693328066939 × 10^13^ for FDB and 2.001039166064110 × 10^13^ for dFDB versus 3.930192909563312 × 10^13^ for SFOA), implying fewer runs dominated by large penalty terms. Overall, the combined evidence from Table 24 and Figure 22 confirms that integrating FDB/dFDB strengthens SFOA on Blending–Pooling by improving both the best attainable objective and the run-to-run reliability under a tight evaluation budget.

#### 4.3.5. Four-Stage Gear Box Problem

The Four-Stage Gear Box problem is a discrete, highly constrained mechanical design task in which a four-stage gear transmission is sized and placed within a bounded gearbox layout while minimizing an overall weight/volume surrogate. The challenge stems from the fact that both *gear sizing*, including tooth numbers and face widths, and *layout decisions*, including shaft coordinates, must be selected from predefined discrete sets, and feasible solutions typically occupy a small portion of the search space. Accordingly, this benchmark is frequently used to evaluate the robustness of metaheuristic optimizers under strict feasibility requirements [64].

**Bounds.** The four-stage gear box problem was implemented with D=22 decision variables. The first eight variables were bounded as gear tooth numbers:

(41) $$6.51\leq x_{i}\leq 76.49,\;i=1,2,\ldots ,8.$$

The next four variables were used as indices for selecting the face-width values and were bounded as

(42) $$0.51\leq x_{i}\leq 4.49,\;i=9,10,11,12.$$

The last ten variables were used as indices for selecting coordinate values and were bounded as

(43) $$0.51\leq x_{i}\leq 9.49,\;i=13,14,\ldots ,22.$$

**Decision variables and discrete decoding.** A candidate solution is represented by a *D*-dimensional vector with D=22, i.e., $x\in R^{22}$. To enforce the discrete nature of the design space, the vector is first integer-coded by rounding x←round(x), and then mapped to physical parameters through fixed lookup sets.

The first eight components encode the pinion and gear tooth numbers for each stage:

(44) $$\begin{array}{c} \left\{N_{p1},N_{g1},N_{p2},N_{g2},N_{p3},N_{g3},N_{p4},N_{g4}\right\}=\left\{x_{1},x_{2},x_{3},x_{4},x_{5},x_{6},x_{7},x_{8}\right\}. \end{array}$$

The face widths are selected via index mapping from the discrete set

(45) P={3.175,5.715,8.255,12.7}mm,

as

(46) $$b_{1}=P\left(x_{9}\right),\;b_{2}=P\left(x_{10}\right),\;b_{3}=P\left(x_{11}\right),\;b_{4}=P\left(x_{12}\right).$$

Similarly, the layout coordinates are chosen from the discrete grid

(47) G={12.7,25.4,38.1,50.8,63.5,76.2,88.9,101.6,114.3}mm,

and decoded as

(48) $$\begin{array}{ccccc} x_{p1} & =G(x_{13}), & \;\; & x_{g1} & =G(x_{14}),\\ x_{g2} & =G(x_{15}), & \;\; & x_{g3} & =G(x_{16}),\\ x_{g4} & =G(x_{17}), & \;\; & y_{p1} & =G(x_{18}),\\ y_{g1} & =G(x_{19}), & \;\; & y_{g2} & =G(x_{20}),\\ y_{g3} & =G(x_{21}), & \;\; & y_{g4} & =G(x_{22}). \end{array}$$

Here, $\left(x_{p1},y_{p1}\right)$ denotes the first pinion shaft location, while $\left(x_{gs},y_{gs}\right)$ corresponds to the gear shaft location of stage s∈{1,2,3,4}.

**Geometric coupling and center distances.** Following the MATLAB implementation, each stage is geometrically characterized by the Euclidean distance between the first pinion center and the gear center of that stage:
  (49) $$c_{s}=\sqrt{{\left(x_{gs}-x_{p1}\right)}^{2}+{\left(y_{gs}-y_{p1}\right)}^{2}},\;s=1,2,3,4.$$
**Objective function.** The objective function used in this paper is identical to the MATLAB implementation and aggregates four stage-wise terms:
  (50) $$\min_{x}\;f\left(x\right)=\frac{\pi }{1000}\sum_{s=1}^{4}b_{s}\;c_{s}^{2}\;\frac{N_{ps}^{2}+N_{gs}^{2}}{{\left(N_{ps}+N_{gs}\right)}^{2}}+10^{6}\;\Phi \left(x\right),$$
  where $b_{s}$ is the face width, $N_{ps}$ and $N_{gs}$ are the pinion and gear tooth numbers of stage *s*, and $c_{s}$ is defined in Equation (49). The second term is the penalty component, where Φ(x)=FourStageGear(x) denotes the penalty value returned by the inequality-constraint function, which evaluates 86 inequality constraints.
**Constraint handling and penalty interpretation.** Feasibility is enforced by a static penalty strategy. For feasible solutions, Φ(x)=0; for infeasible solutions, Φ(x)>0, and the objective in Equation (50) is increased by $10^{6}\Phi \left(x\right)$. In the MATLAB implementation, all decision variables are first rounded to the nearest integer before objective and constraint evaluation. The same decoding procedure, bounds, objective function, 86 inequality constraints, and penalty settings were applied consistently to all compared algorithms. Since the inequality violation is internally multiplied by $10^{6}$, the final penalized objective corresponds to
  (51) $$F_{\mathrm{pen}}\left(x\right)=f_{0}\left(x\right)+10^{12}CV_{\mathrm{ineq}}\left(x\right),$$
  where $CV_{\mathrm{ineq}}\left(x\right)$ is defined in Equation (26).
**Problem size summary.** With the above encoding, the problem has D=22 discrete decision variables: 8 tooth number variables, 4 face-width indices, and 10 coordinate indices. The constraint set comprises M=86 inequality constraints, which are aggregated in Φ(x)=FourStageGear(x) and handled via the penalty term in Equation (50).

Table 25 reports the best design variables obtained by FDBSFOA and dFDBSFOA for the four-stage gear box problem. The reported values are the best optimizer outputs before discrete decoding. In the four-stage gear box implementation, these values are rounded and mapped to the predefined gear tooth, face-width, and coordinate sets before objective and constraint evaluation. The corresponding best penalized objective values are 36.5652 for FDBSFOA and 36.4779 for dFDBSFOA, indicating that dFDBSFOA obtains a slightly better best-found design under the same decoding and penalty settings.

**Figure 23:**
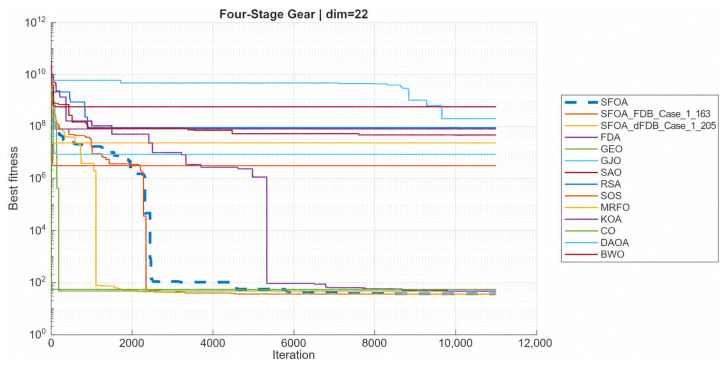
Convergence curves (log-scale) on the Four-Stage Gear problem, D=22, over 21 runs.

Results and discussion. Table 26 summarizes the statistical results on the Four-Stage Gear problem with D=22 over 21 independent runs, and Figure 23 presents the best-so-far convergence profiles on a log scale. Overall, the SFOA-based variants exhibit a clear advantage in navigating the penalty-dominated infeasible regions induced by strict constraints and discrete decoding. In terms of best-achieved solution quality, SFOA_dFDB_Case_1_205 attains the lowest objective value $\left(3.62515\times 10^{1}\right)$, indicating the strongest peak performance. Moreover, its worst value is dramatically smaller than that of the baseline SFOA, suggesting that dFDB reduces the likelihood of severely penalized outcomes. From a robustness perspective, SFOA_FDB_Case_1_163 yields the lowest standard deviation $\left(3.41335\times 10^{6}\right)$ and a low median $\left(6.46431\times 10^{1}\right)$, implying comparatively more stable run-to-run behavior under the discrete feasibility constraints. By contrast, the baseline SFOA shows much larger variability and a substantially higher worst-case value $\left(1.62189\times 10^{7}\right)$, indicating that a non-negligible fraction of runs stagnate in infeasible or poorly conditioned regions. The convergence curves in Figure 23 corroborate these findings: the enhanced variants achieve pronounced objective reductions earlier and maintain steady refinement, whereas several competing algorithms stagnate at considerably higher fitness levels over long iteration horizons. Overall, the results confirm that incorporating dFDB improves the best attainable solution quality, while FDB enhances the consistency of SFOA on this discrete, highly constrained design task.

### 4.4. Managerial Insights

Managerial insights are provided to help practitioners and decision makers translate the numerical evidence into actionable guidance for real-world optimization workflows, especially when objective evaluations are expensive and design constraints are strict. The proposed FDBSFOA and dFDBSFOA variants were developed to improve the exploration–exploitation balance of SFOA and to mitigate premature convergence under challenging landscapes, which is frequently observed in practical engineering design tasks.

When to prefer FDBSFOA versus dFDBSFOA.

The experimental evidence suggests a clear usage guideline: for low- to medium-dimensional continuous design problems, FDB-based variants provide stable and consistent improvement over the baseline, whereas for high-dimensional and complex landscapes, especially hybrid and composition-type problems, dFDB-based variants provide more pronounced gains in convergence speed and final solution quality. This implies that engineering teams dealing with large-scale parameter calibration, data-driven surrogate tuning, or large continuous design vectors can prioritize dFDBSFOA as a first-choice solver, whereas FDBSFOA can serve as a lightweight enhancement when dimensionality is limited.

Robustness as a practical performance criterion.

In many engineering organizations, repeatability is as important as the best single objective value. In the engineering design experiments, SFOA, FDBSFOA, and dFDBSFOA often reached the same or nearly the same best-known optima, while some enhanced variants yielded lower standard deviations, indicating more robust outcomes across runs. This is practically valuable because it reduces the number of reruns required to obtain a reliable feasible design, shortens the verification cycle, and lowers the risk of decision-making based on a single lucky run.

Implications for engineering design management.

From a managerial standpoint, the numerical results support three operational recommendations. First, for black-box engineering design tasks where evaluations are costly, it is beneficial to allocate the computational budget to algorithms that converge faster and exhibit lower run-to-run variability; both aspects directly influence time-to-decision. Second, teams can adopt a two-stage workflow: use a dFDBSFOA configuration for global exploration and rapid convergence, then refine feasibility and local tuning in the later iterations, or by a local search method if available, once constraints become active. Third, because the methods are implemented and tested in a standard MATLAB environment on commodity hardware, they are compatible with typical engineering analytics pipelines without requiring specialized computing infrastructure.

Limitations and boundary of applicability.

The proposed mechanisms are validated primarily on continuous CEC benchmark suites and a set of classical constrained engineering design problems, including both continuous and discrete/integer-coded cases. Nevertheless, broader applicability to highly noisy, dynamic, simulation-driven, large-scale mixed-integer, or multi-objective problems may require additional adaptations, such as specialized discretization, noise-handling, and constraint-handling strategies. Moreover, although the proposed mechanisms improve robustness and mitigate premature convergence, metaheuristic search remains stochastic; therefore, practitioners should still use multiple independent runs and report dispersion measures rather than relying solely on a single best value.

Interpretation of penalized engineering results.

The engineering-design statistics should be interpreted together with the penalty formulation in Equations (28) and (30). Since the reported values correspond to penalized objectives, extremely large Worst, Mean, Std, or IQR values indicate that the corresponding algorithm generated constraint-violating final solutions in some runs and was therefore strongly penalized. Conversely, the lower dispersion observed for FDBSFOA and dFDBSFOA indicates more stable behavior under the same constraint-handling setting. This provides an indirect feasibility-related interpretation of the reported engineering-design statistics without changing the original experimental protocol.

Practical takeaway.

Overall, FDBSFOA and dFDBSFOA can be positioned as practical and competitive global optimizers for constrained engineering design and benchmark optimization, with an emphasis on improved convergence behavior in complex and high-dimensional settings and enhanced robustness in engineering design runs, two criteria that directly matter for engineering teams responsible for reliable design decisions under limited evaluation budgets.

## 5. Conclusions

This paper proposes two enhanced variants of the Starfish Optimization Algorithm (SFOA), namely FDBSFOA and dFDBSFOA, by embedding a fitness–distance-aware selection mechanism to explicitly balance exploration and exploitation. The core idea is to complement fitness-based intensification with a diversity-preserving distance term so that the population can both avoid premature convergence and contract effectively near promising regions. In dFDBSFOA, this balance is controlled through a scheduled coefficient updated periodically during the search, yielding a systematic transition from exploration-oriented behavior to exploitation-oriented refinement without requiring extensive manual tuning.

Comprehensive experiments were conducted on CEC2017, CEC2020, and CEC2022 benchmark suites under a unified evaluation budget (MaxFEs= 10,000×D) and 21 independent runs, and were complemented by constrained engineering design problems (with MaxFEs>1000). The comparative analysis, supported by nonparametric statistical tests (Friedman ranking and Wilcoxon signed-rank, α=0.05), demonstrates that the proposed mechanisms particularly the dynamic scheduling in dFDBSFOA provide more stable performance and improved convergence behavior compared to the baseline SFOA and fixed selection structures. Although the added selection step introduces additional distance computations, the overhead remains practical relative to the overall evaluation budget, and the methods can be used as drop-in alternatives to SFOA under identical stopping rules and boundary handling (random re-initialization within bounds).

Despite these strengths, the current study is limited to continuous, single-objective, stationary optimization. The behavior on discrete, dynamic, or multi-objective formulations has not been investigated, and the impact of the scheduling bounds and update frequency may vary across problem classes. Moreover, while the benchmarks cover multiple dimensions, very large-scale settings beyond the tested ranges may require additional efficiency considerations due to distance-related computations and the growth of the search space.

Future work will focus on three concrete directions. Firstly, the proposed selection mechanism will be extended to multi-objective, constrained multi-objective, and discrete scenarios by redesigning the fitness distance score to operate on Pareto dominance, constraint handling rules, and diversity indicators, and by incorporating discrete neighborhood operators where appropriate. Secondly, motivated by the observation that distance-to-best may be insufficient to characterize population dispersion, a fully dedicated distance-metric ablation study. Third, building on the CFG sensitivity study, we will develop fully self-adaptive schedules that learn, online, the scheduling bounds for the fitness and distance/diversity weights and the update frequency, and we will evaluate scalability on higher-dimensional and large-scale problems (including dynamic test settings). This direction will also include efficient implementations that reduce the overhead of distance calculations (e.g., partial updates, vectorization, or approximate neighbor queries) while preserving solution quality. 

## Figures and Tables

**Table 1:** Taxonomy of metaheuristics and reference mapping used in this paper, by family.

MOA Category	Core Update Principle	References
General MOA motivation	Derivative-free global search; exploration–exploitation control	[7,8,9]
Evolutionary algorithms	Variation + selection (mutation/crossover, survival)	GA [11], DE [13]
Swarm intelligence	Collective interaction rules; social learning and guidance	PSO [12], GWO [15], HHO [17]
Physics/mathematics inspired	Equilibrium/dynamics-inspired transitions	EO [16]
Human/learning inspired	Teacher–learner learning phases; limited control parameters	TLBO [14]
Bio-inspired (recent)	Nature-inspired operators and recently proposed bio-inspired search mechanisms	SFOA [25], POA [39], FOSSA [40], LFS [26], WUTP [27]
Diversity analysis/preservation	Dispersion metrics and diversity-aware robustness strategies	[18,19,41]
Fitness–distance-aware selection	Jointly scores quality + spatial diversity (fixed/dynamic weighting)	FDB [20], dFDB [21]
Recent enhancement trends (incl. 2025)	Adaptive control, hybridization, learning-assisted strategies, and MOA-related application frameworks validated on practical optimization problems	[28,29,30,31,32,37,38]

**Table 2:** Key methods cited in related works: publication year, type, and main search principle.

Method	Year	Type	Main Idea (One Line; What It Is Built on)	Ref.
GA	1992	Core MOA (EA)	Evolution via variation and selection operators	[11]
PSO	1995	Core MOA (SI)	Social learning using personal/global best guidance	[12]
DE	1997	Core MOA (EA)	Differential mutation and recombination for real-valued search	[13]
TLBO	2011	Core MOA (Human/learning)	Teacher and learner phases with limited parameters	[14]
GWO	2014	Core MOA (SI)	Leadership hierarchy and encircling/hunting-inspired updates	[15]
HHO	2019	Core MOA (SI)	Switching exploration/exploitation through besiege strategies	[17]
EO	2020	Core MOA (Physics-inspired)	Equilibrium-state modeling for search transitions	[16]
FDB	2020	Selection mechanism	Fitness + distance-to-best scoring for ranking/survival	[20]
dFDB	2022	Selection mechanism	Dynamic scheduling of the fitness–distance trade-off	[21]
Population diversity metric	2022	Diversity analysis	Quantifies dispersion to support robustness and avoid crowding	[18]
SFOA	2025	Core MOA (Bio-inspired)	Starfish-inspired search; compared against a large pool of optimizers	[25]
Lionfish Search Algorithm (LFS)	2025	Core MOA (Bio-inspired)	A newly proposed nature-inspired optimizer based on lionfish predatory behavior	[26]
Water Uptake and Transport in Plants algorithm	2025	Core MOA (Bio-inspired)	Plant water-transport-inspired search dynamics for numerical optimization	[27]
Hybrid Snake Optimizer with Crisscross Learning	2025	Hybrid/improved MOA	Snake optimizer strengthened by crisscross and lens-imaging learning for broader search coverage	[28]
RL-based improved Snake Optimizer	2025	Adaptive/learning-assisted MOA	Q-learning-guided parameter adaptation to improve real-world engineering search	[29]
Random walk-based GOOSE	2025	Core MOA (Engineering-oriented)	A random-walk-enhanced GOOSE search strategy for engineering structural design problems	[30]
Hierarchical MRFO for trust-aware routing	2025	MOA-assisted framework	Hierarchical manta-ray-foraging optimization embedded into secure MANET routing with selfish-node detection	[31]
Emerging metaheuristics benchmark study	2025	Comparative study	Comparative evaluation of recent metaheuristics on vehicle routing problems	[37]
Hybrid SLM+PTS+RNN	2025	MOA-assisted framework	Hybrid optimization/learning framework for reducing PAPR in UFMC/B5G communication systems	[32]
Tournament TLBO	2025	TLBO variant	Adjusts selection pressure in TLBO via tournament selection logic	[38]

**Table 3:** CFG definitions for the scheduled distance/diversity weight coefficient β(t)=1−α(t).

CFG	$\beta_{\max}$	$\beta_{\min}$	Interpretation
CFG1	0.2	0.0	Fitness-dominant schedule; favors exploitation and may reduce diversity.
CFG2	0.6	0.0	Balanced schedule; close to the main experimental line.
CFG3	0.8	0.2	Diversity-preserving schedule; maintains stronger distance contribution during the search.

**Table 4:** SFOA equations and candidate selection using FDB and dFDB.

SFOA Case	Equation (23)	Equation (24)	Equation (25)
FDB_Case_1_136	-	A	E
FDB_Case_1_163	-	A	A-B-C
FDB_Case_1_183	-	A	B-C-D-E
FDB_Case_1_205	-	A	A-B-D-E
dFDB_Case_1_110	A	A	-
dFDB_Case_1_159	-	A	A-C-E
dFDB_Case_1_167	-	A	A-B-E
dFDB_Case_1_171	-	A	B-C-E
dFDB_Case_1_179	-	A	A-D-E
dFDB_Case_1_183	-	A	C-D-E
dFDB_Case_1_205	-	A	A-B-D-E
dFDB_Case_1_209	-	A	B-C-D-E

**Table 5:** Normalized runtime complexity on CEC2022 F1 using $\mathrm{Complexity}=\left(\bar{T_{2}}-T_{1}\right)/T_{0}$ for D∈{10,20}.

Algorithm	F1 (D = 10)	F1 (D = 20)	$T_{0}$ (s)	$T_{1}$ (s)
Reference timing: $T_{0}=0.0057$; $T_{1}=0.0931$ (D=10) and $T_{1}=0.1195$ (D=20).				
SFOA	12.8753	13.9868	0.0057	0.0931 (D = 10) 0.1195 (D = 20)
SFOA_FDB_Case_1_136	14.7151	16.7617		
SFOA_FDB_Case_1_163	14.3488	16.5580		
SFOA_FDB_Case_1_183	14.7474	16.6989		
SFOA_FDB_Case_1_205	14.3245	16.9886		
SFOA_dFDB_Case_1_110	14.5504	17.1039		
SFOA_dFDB_Case_1_159	15.8690	17.0304		
SFOA_dFDB_Case_1_167	14.4831	16.7029		
SFOA_dFDB_Case_1_171	14.6372	16.5169		
SFOA_dFDB_Case_1_179	14.7997	16.2978		
SFOA_dFDB_Case_1_183	14.6547	15.8763		
SFOA_dFDB_Case_1_205	14.6946	16.8686		
SFOA_dFDB_Case_1_209	14.5013	16.9076		

**Table 6:** Normalized runtime complexity on CEC2022 F7 using Complexity $=\left({\bar{T}}_{2}-T_{1}\right)/T_{0}$ for D∈{10,20}.

Algorithm	F7 (D = 10)	F7 (D = 20)	$T_{0}$ (s)	$T_{1}$ (s)
Reference timing: $T_{0}=0.0079$; $T_{1}=0.3418$ (D = 10) and $T_{1}=0.6112$ (D = 20).				
SFOA	12.0742	13.3680	0.0079	0.3418 (D = 10) 0.6112 (D = 20)
SFOA_FDB_Case_1_136	13.2465	15.4422		
SFOA_FDB_Case_1_163	12.9729	15.7804		
SFOA_FDB_Case_1_183	13.2382	15.5988		
SFOA_FDB_Case_1_205	13.6367	16.4888		
SFOA_dFDB_Case_1_110	13.6445	16.4142		
SFOA_dFDB_Case_1_159	13.3257	16.8812		
SFOA_dFDB_Case_1_167	13.4404	16.0781		
SFOA_dFDB_Case_1_171	13.3401	16.4764		
SFOA_dFDB_Case_1_179	13.1446	15.9023		
SFOA_dFDB_Case_1_183	13.4667	16.1880		
SFOA_dFDB_Case_1_205	13.2862	16.5422		
SFOA_dFDB_Case_1_209	13.3739	15.8172		

**Table 7:** Normalized runtime complexity on CEC2022 F12 using Complexity $=\left({\bar{T}}_{2}-T_{1}\right)/T_{0}$ for D∈{10,20}.

Algorithm	F12 (D = 10)	F12 (D = 20)	$T_{0}$ (s)	$T_{1}$ (s)
Reference timing: $T_{0}=0.0079$; $T_{1}=0.5035$ (D = 10) and $T_{1}=0.9941$ (D = 20).				
SFOA	8.5204	13.2482	0.0079	0.5035 (D = 10) 0.9941 (D = 20)
SFOA_FDB_Case_1_136	9.9157	16.3142		
SFOA_FDB_Case_1_163	9.9935	14.6783		
SFOA_FDB_Case_1_183	9.6931	15.5500		
SFOA_FDB_Case_1_205	9.8038	14.4807		
SFOA_dFDB_Case_1_110	9.4144	15.4616		
SFOA_dFDB_Case_1_159	9.7390	14.6190		
SFOA_dFDB_Case_1_167	9.8253	14.6986		
SFOA_dFDB_Case_1_171	9.8043	15.2926		
SFOA_dFDB_Case_1_179	9.8701	15.7282		
SFOA_dFDB_Case_1_183	9.7185	15.1129		
SFOA_dFDB_Case_1_205	9.8855	15.2681		
SFOA_dFDB_Case_1_209	11.0993	15.2347		

**Table 8:** Normalized runtime complexity on CEC2017 F1 using $\mathrm{Complexity}=\left({\bar{T}}_{2}-T_{1}\right)/T_{0}$ for D∈{30,50,100}.

Algorithm	F1 (D = 30)	F1 (D = 50)	F1 (D = 100)
Reference timing:	$T_{0}=0.0192$ s; $T_{1}=0.6344$ s (D = 30), 1.9429 s (D = 50), and 12.4469 s (D = 100).		
SFOA	2553.7454	5668.8771	30,973.4941
SFOA_FDB_Case_1_136	2311.8538	6205.8611	31,904.2944
SFOA_FDB_Case_1_163	2357.6296	5874.3180	31,610.0012
SFOA_FDB_Case_1_183	2344.2346	5979.9190	31,652.1819
SFOA_FDB_Case_1_205	2310.0948	6014.3416	31,716.5242
SFOA_dFDB_Case_1_110	2363.3140	6071.5554	31,611.4051
SFOA_dFDB_Case_1_159	2299.7345	5859.4910	31,569.4293
SFOA_dFDB_Case_1_167	2358.7047	5963.0759	31,562.9049
SFOA_dFDB_Case_1_171	2314.3592	5810.5102	31,651.3937
SFOA_dFDB_Case_1_179	2461.8704	5936.5843	37,259.8021
SFOA_dFDB_Case_1_183	3344.1798	6030.5215	32,115.3956
SFOA_dFDB_Case_1_205	2303.2963	5859.3908	31,551.1577
SFOA_dFDB_Case_1_209	3488.8539	6414.5635	31,482.4919

**Table 9:** Normalized runtime complexity on CEC2017 F15 using $\mathrm{Complexity}=\left(\bar{T_{2}}-T_{1}\right)/T_{0}$ for D∈{50,100}.

Algorithm	F15 (D = 50)	F15 (D = 100)
Reference timing:	$T_{0}=0.025995$ s; $T_{1}=2.798419$ s (D = 50), and 16.993419 s (D = 100).	
SFOA	8507.0127	29,224.5593
SFOA_FDB_Case_1_136	6474.8426	34,911.8853
SFOA_FDB_Case_1_163	6363.7791	39,003.6706
SFOA_FDB_Case_1_183	6121.5463	37,264.2630
SFOA_FDB_Case_1_205	7112.2244	35,489.5582
SFOA_dFDB_Case_1_110	7088.7104	51,883.7893
SFOA_dFDB_Case_1_159	6482.6799	29,688.9328
SFOA_dFDB_Case_1_167	6030.5743	29,396.1554
SFOA_dFDB_Case_1_171	5988.6028	29,698.6290
SFOA_dFDB_Case_1_179	5943.8568	29,853.7862
SFOA_dFDB_Case_1_183	5951.6787	29,984.6756
SFOA_dFDB_Case_1_205	6037.1305	30,060.3154
SFOA_dFDB_Case_1_209	5881.5595	29,887.0821

**Table 10:** Normalized runtime complexity on CEC2017 F7 using $\mathrm{Complexity}=\left(\bar{T_{2}}-T_{1}\right)/T_{0}$ for D∈{30,50,100}.

Algorithm	F7 (D = 30)	F7 (D = 50)	F7 (D = 100)
Reference timing:	$T_{0}=0.022468$ s; $T_{1}=1.190835$ s (D = 30), 3.593922 s (D = 50), and 18.847213 s (D = 100).		
SFOA	3095.4164	8956.5540	38,155.7436
SFOA_FDB_Case_1_136	3413.5751	9315.5010	40,123.4110
SFOA_FDB_Case_1_163	3168.8806	8692.5039	44,017.3214
SFOA_FDB_Case_1_183	3593.8938	10,489.4115	46,900.6225
SFOA_FDB_Case_1_205	4156.9478	9045.5042	49,199.3362
SFOA_dFDB_Case_1_110	3913.9870	10,577.1648	48,941.2099
SFOA_dFDB_Case_1_159	3692.9591	9732.6155	41,180.7460
SFOA_dFDB_Case_1_167	3368.5679	9312.8113	41,280.5429
SFOA_dFDB_Case_1_171	3337.2614	9267.9142	41,211.4695
SFOA_dFDB_Case_1_179	3342.6256	9221.1877	41,041.4238
SFOA_dFDB_Case_1_183	3363.1144	9248.9101	42,649.9500
SFOA_dFDB_Case_1_205	3376.1591	9240.6010	41,308.9073
SFOA_dFDB_Case_1_209	3367.0947	9313.3854	41,250.0372

**Table 11:** Summary of the CEC2017 test functions. Here, $F_{i}^{*}$ denotes the known global optimum value of the *i*th benchmark function.

No.	Function	$F_{i}^{*}=F_{i}\left(x^{*}\right)$
**Unimodal Functions**		
1	Shifted and Rotated Bent Cigar Function	100
2	Shifted and Rotated Zakharov Function	200
**Simple Multimodal Functions**		
3	Shifted and Rotated Rosenbrock’s Function	300
4	Shifted and Rotated Rastrigin’s Function	400
5	Shifted and Rotated Expanded Schaffer’s F6 Function	500
6	Shifted and Rotated Lunacek Bi–Rastrigin Function	600
7	Shifted and Rotated Non-Continuous Rastrigin’s Function	700
8	Shifted and Rotated Levy Function	800
9	Shifted and Rotated Schwefel’s Function	900
**Hybrid Functions**		
10	Hybrid Function 1 (N=3)	1000
11	Hybrid Function 2 (N=3)	1100
12	Hybrid Function 3 (N=3)	1200
13	Hybrid Function 4 (N=4)	1300
14	Hybrid Function 5 (N=4)	1400
15	Hybrid Function 6 (N=4)	1500
16	Hybrid Function 7 (N=5)	1600
17	Hybrid Function 8 (N=5)	1700
18	Hybrid Function 9 (N=5)	1800
19	Hybrid Function 10 (N=6)	1900
**Composition Functions**		
20	Composition Function 1 (N=3)	2000
21	Composition Function 2 (N=3)	2100
22	Composition Function 3 (N=4)	2200
23	Composition Function 4 (N=4)	2300
24	Composition Function 5 (N=5)	2400
25	Composition Function 6 (N=5)	2500
26	Composition Function 7 (N=6)	2600
27	Composition Function 8 (N=6)	2700
28	Composition Function 9 (N=3)	2800
29	Composition Function 10 (N=3)	2900

**Table 12:** Summary of the CEC2020 test suite. Here, $F_{i}^{*}$ denotes the known global optimum value of the *i*th benchmark function.

Category	Function Description	$F_{i}^{*}=F\left(x^{*}\right)$
Unimodal	1. Shifted and Rotated Bent Cigar Function	100
Basic	2. Shifted and Rotated Schwefel’s Function	1100
	3. Shifted and Rotated Lunacek Bi–Rastrigin Function	700
	4. Expanded Rosenbrock’s plus Griewangk’s Function	1900
Hybrid	5. Hybrid Function 1 (N=3)	1700
	6. Hybrid Function 2 (N=4)	1600
	7. Hybrid Function 3 (N=5)	2100
Composition	8. Composition Function 1 (N=3)	2200
	9. Composition Function 2 (N=4)	2400
	10. Composition Function 3 (N=5)	2500
Search range:		${\left[-100,100\right]}^{D}$

**Table 13:** Summary of the CEC2022 test suite. Here, $F_{i}^{*}$ denotes the known global optimum value of the *i*th benchmark function.

Category	Function Description	$F_{i}^{*}$
Unimodal	1. Shifted and Fully Rotated Zakharov Function	300
Basic	2. Shifted and Fully Rotated Rosenbrock’s Function	400
	3. Shifted and Fully Rotated Expanded Schaffer’s $f_{6}$ Function	600
	4. Shifted and Fully Rotated Non-Continuous Rastrigin’s Function	800
	5. Shifted and Fully Rotated Levy Function	900
Hybrid	6. Hybrid Function 1 (N=3)	1800
	7. Hybrid Function 2 (N=6)	2000
	8. Hybrid Function 3 (N=5)	2200
Composition	9. Composition Function 1 (N=5)	2300
	10. Composition Function 2 (N=4)	2400
	11. Composition Function 3 (N=5)	2600
	12. Composition Function 4 (N=6)	2700
Search range:		${\left[-100,100\right]}^{D}$

**Table 14:** Wilcoxon pairwise comparison results between SFOA and its variants on CEC2022.

vs. SFOA (+/=/−)	Dimension = 10	Dimension = 20
SFOA_FDB_Case_1_136	2/7/3	6/5/1
SFOA_FDB_Case_1_163	2/7/3	5/7/0
SFOA_FDB_Case_1_183	2/8/2	7/5/0
SFOA_FDB_Case_1_205	3/6/3	7/5/0
SFOA_dFDB_Case_1_110	1/10/1	5/7/0
SFOA_dFDB_Case_1_159	2/9/1	3/9/0
SFOA_dFDB_Case_1_167	1/10/1	5/7/0
SFOA_dFDB_Case_1_171	2/9/1	6/6/0
SFOA_dFDB_Case_1_179	1/10/1	5/7/0
SFOA_dFDB_Case_1_183	2/9/1	5/7/0
SFOA_dFDB_Case_1_205	1/10/1	6/6/0
SFOA_dFDB_Case_1_209	2/9/1	5/7/0

Note: +/=/− denote the number of functions for which the variant performed significantly better/similarly/worse than the standard SFOA.

**Table 15:** Wilcoxon pairwise comparison results between the standard SFOA and its variants on the CEC2020 test suite.

vs. SFOA (+/=/−)	Dimension = 30	Dimension = 50	Dimension = 100
SFOA_FDB_Case_1_136	5/5/0	5/5/0	1/9/0
SFOA_FDB_Case_1_163	4/6/0	3/7/0	2/8/0
SFOA_FDB_Case_1_183	4/6/0	4/6/0	2/8/0
SFOA_FDB_Case_1_205	3/7/0	3/7/0	5/5/0
SFOA_dFDB_Case_1_110	7/3/0	5/5/0	4/6/0
SFOA_dFDB_Case_1_159	5/4/1	4/6/0	3/7/0
SFOA_dFDB_Case_1_167	5/4/1	5/5/0	3/7/0
SFOA_dFDB_Case_1_171	4/5/1	5/5/0	3/7/0
SFOA_dFDB_Case_1_179	5/4/1	6/4/0	3/7/0
SFOA_dFDB_Case_1_183	4/5/1	3/7/0	3/7/0
SFOA_dFDB_Case_1_205	4/5/1	5/5/0	3/7/0
SFOA_dFDB_Case_1_209	4/5/1	6/4/0	3/7/0

Note: +/=/− denote the number of functions for which the variant performed significantly better/similarly/worse than the standard SFOA.

**Table 16:** Wilcoxon pairwise comparison results between SFOA and its variants on CEC2017.

vs. SFOA (+/=/−)	Dim = 10	Dim = 30	Dim = 50	Dim = 100
SFOA_FDB_Case_1_136	7/20/2	16/13/0	20/8/1	14/13/2
SFOA_FDB_Case_1_163	7/20/2	16/13/0	15/13/1	14/14/1
SFOA_FDB_Case_1_183	7/19/3	14/15/0	18/9/2	13/16/0
SFOA_FDB_Case_1_205	9/17/3	16/13/0	15/13/1	14/15/0
SFOA_dFDB_Case_1_110	5/21/3	18/10/1	22/5/2	19/9/1
SFOA_dFDB_Case_1_159	6/20/3	18/10/1	21/7/1	16/13/0
SFOA_dFDB_Case_1_167	8/17/4	19/9/1	21/8/0	19/8/2
SFOA_dFDB_Case_1_171	7/19/3	17/11/1	21/7/1	17/11/1
SFOA_dFDB_Case_1_179	6/19/4	21/7/1	23/5/1	19/9/1
SFOA_dFDB_Case_1_183	8/19/2	17/11/1	21/7/1	18/10/1
SFOA_dFDB_Case_1_205	7/18/4	19/9/1	22/7/0	17/11/1
SFOA_dFDB_Case_1_209	7/20/2	17/11/1	22/6/1	19/10/0

Note: +/=/− denote the number of functions for which the variant performed significantly better/similarly/worse than the standard SFOA.

**Table 17:** Friedman ranking results of SFOA and FDBSFOA variants on the CEC2020 and CEC2022 benchmark suites under different dimensional settings. Lower Friedman rank indicates better overall performance.

Algorithm	CEC2020-100D	CEC2020-50D	CEC2020-30D	CEC2022-10D	CEC2022-20D
SFOA	8.485714	3.578571	3.609524	2.910714	3.630952
SFOA_FDB_CASE_1_136	6.995238	2.840476	2.814286	3.065476	2.855159
SFOA_FDB_CASE_1_163	7.535714	2.907143	2.816667	3.079365	2.821429
SFOA_FDB_CASE_1_183	7.266667	2.792857	2.888095	3.035714	2.813492
SFOA_FDB_CASE_1_205	7.052381	2.880952	2.871429	2.908730	2.878968

**Table 18:** Friedman ranking results of SFOA and dFDBSFOA variants on the CEC2020 and CEC2022 benchmark suites under different dimensional settings. Lower Friedman rank indicates better overall performance.

Algorithm	CEC2020-100D	CEC2020-50D	CEC2020-30D	CEC2022-10D	CEC2022-20D
SFOA	8.485714	6.404762	6.000000	5.045635	5.980159
SFOA_DFDB_CASE_1_110	6.076190	4.583333	4.319048	5.001984	4.878968
SFOA_DFDB_CASE_1_159	6.547619	4.809524	4.911905	5.051587	4.950397
SFOA_DFDB_CASE_1_167	6.885714	4.821429	4.764286	5.097222	4.898810
SFOA_DFDB_CASE_1_171	6.514286	4.900000	4.950000	4.783730	4.908730
SFOA_DFDB_CASE_1_179	6.785714	4.814286	4.947619	5.043651	4.863095
SFOA_DFDB_CASE_1_183	7.038095	5.142857	5.126190	4.954365	4.966270
SFOA_DFDB_CASE_1_205	7.000000	4.892857	4.985714	4.986111	4.805556
SFOA_DFDB_CASE_1_209	6.816667	4.630952	4.995238	5.035714	4.748016

**Table 19:** Post-hoc comparison of FDB/dFDB-based SFOA variants against the baseline SFOA on CEC2017 with 50 dim. Lower average rank indicates better performance.

Algorithm	Avg. Rank	Raw *p*	Bonf. *p*	Holm *p*	Finner *p*
SFOA	11.8966	–	–	–	–
SFOA_FDB_Case_1_136	7.5517	$2.1543\times 10^{-5}$	$2.5852\times 10^{-4}$	$6.4630\times 10^{-5}$	$2.5852\times 10^{-5}$
SFOA_FDB_Case_1_163	8.1724	$2.7119\times 10^{-4}$	$3.2543\times 10^{-3}$	$2.7119\times 10^{-4}$	$2.7119\times 10^{-4}$
SFOA_FDB_Case_1_183	7.2414	$5.3211\times 10^{-6}$	$6.3854\times 10^{-5}$	$2.1285\times 10^{-5}$	$7.0948\times 10^{-6}$
SFOA_FDB_Case_1_205	7.8103	$6.4588\times 10^{-5}$	$7.7505\times 10^{-4}$	$1.2918\times 10^{-4}$	$7.0459\times 10^{-5}$
SFOA_dFDB_Case_1_110	4.0000	$1.1546\times 10^{-14}$	$1.3856\times 10^{-13}$	$1.3856\times 10^{-13}$	$1.3856\times 10^{-13}$
SFOA_dFDB_Case_1_159	5.1379	$3.8847\times 10^{-11}$	$4.6616\times 10^{-10}$	$4.2732\times 10^{-10}$	$2.3308\times 10^{-10}$
SFOA_dFDB_Case_1_167	6.9828	$1.5507\times 10^{-6}$	$1.8609\times 10^{-5}$	$8.5514\times 10^{-6}$	$2.4433\times 10^{-6}$
SFOA_dFDB_Case_1_171	6.3448	$5.6882\times 10^{-8}$	$6.8258\times 10^{-7}$	$4.5505\times 10^{-7}$	$1.4120\times 10^{-7}$
SFOA_dFDB_Case_1_179	6.6207	$2.4880\times 10^{-7}$	$2.9855\times 10^{-6}$	$1.7416\times 10^{-6}$	$4.9759\times 10^{-7}$
SFOA_dFDB_Case_1_183	6.3103	$4.7066\times 10^{-8}$	$5.6479\times 10^{-7}$	$4.2359\times 10^{-7}$	$1.4120\times 10^{-7}$
SFOA_dFDB_Case_1_205	6.9655	$1.4252\times 10^{-6}$	$1.7103\times 10^{-5}$	$8.5514\times 10^{-6}$	$2.4433\times 10^{-6}$
SFOA_dFDB_Case_1_209	5.9655	$6.6625\times 10^{-9}$	$7.9950\times 10^{-8}$	$6.6625\times 10^{-8}$	$2.6650\times 10^{-8}$

**Table 20:** Constraint-handling and bound summary of the engineering design problems used in this study.

Problem	Dim.	Constraint Type	Effective Penalty	Bounds/Discrete Handling
Tension/compression spring	3	4 inequalities	$f_{0}\left(x\right)+10^{12}CV_{\mathrm{ineq}}\left(x\right)$	$x_{1}\in \left[0.05,2.0\right]$, $x_{2}\in \left[0.25,1.3\right]$, $x_{3}\in \left[2.0,15.0\right]$. Continuous variables.
Pressure vessel	4	4 inequalities	$f_{0}\left(x\right)+10^{12}CV_{\mathrm{ineq}}\left(x\right)$	$x_{1},x_{2}\in \left[0.0625,6.1875\right]$, $x_{3},x_{4}\in \left[10,200\right]$. Continuous bounded implementation; no additional rounding of $x_{1},x_{2}$.
Cantilever beam	5	1 inequality	$f_{0}\left(x\right)+10^{12}CV_{\mathrm{ineq}}\left(x\right)$	$x_{i}\in \left[0.01,100\right]$, i=1,…,5. Continuous variables.
Blending–Pooling–Separation	38	32 equality-type constraints	$f_{0}\left(x\right)+10^{12}CV_{\mathrm{eq}}\left(x\right)$, tolerance $10^{-6}$	$lb=0_{1\times 38}$; upper bounds are given in Equation (40). Continuous variables.
Four-stage gear box	22	86 inequalities	$f_{0}\left(x\right)+10^{12}CV_{\mathrm{ineq}}\left(x\right)$	Integer/discrete decoding. $x_{1}$–$x_{8}$ are rounded gear tooth numbers; $x_{9}$–$x_{12}$ and $x_{13}$–$x_{22}$ are rounded indices for predefined face-width and coordinate sets.

**Table 21:** Statistical results on the Cantilever Beam design problem (dim = 5).

Algorithm	Best	Worst	Mean	Std	Median	IQR
SFOA	3.679444	3.679444	3.679444	$9.10\;\times \;10^{-16}$	3.679444	0
SFOA_FDB_Case_1_163	3.679444	3.679444	3.679444	$9.10\;\times \;10^{-16}$	3.679444	0
SFOA_dFDB_Case_1_205	3.679444	3.679444	3.679444	$9.10\;\times \;10^{-16}$	3.679444	0
FDA	3.818749	4.936087	4.137240	0.275003	4.051576	0.351296
GEO	3.760000	4.467143	4.008936	0.183540	3.966383	0.186165
GJO	3.793123	5.349057	4.344912	0.397786	4.336504	0.473646
SAO	$3.25\;\times \;10^{12}$	$1.37125\;\times \;10^{14}$	$3.32039\;\times \;10^{13}$	$3.11253\;\times \;10^{13}$	$2.45\;\times \;10^{13}$	$3.84127\;\times \;10^{13}$
RSA	4.310000	$5.75\;\times \;10^{12}$	$1.41667\;\times \;10^{12}$	$2.08467\;\times \;10^{12}$	5.145714	$4.00\;\times \;10^{12}$
SOS	3.708346	4.276667	3.867798	0.167437	3.785524	0.263530
MRFO	3.679444	4.392609	3.842307	0.204484	3.757368	0.181625
KOA	3.702850	4.228587	3.796215	0.115505	3.759340	0.076877
CO	3.742898	4.300476	3.960717	0.146708	3.928462	0.202467
DAOA	6.741579	$1.875\;\times \;10^{12}$	$1.40476\;\times \;10^{12}$	$8.11928\;\times \;10^{11}$	$1.875\;\times \;10^{12}$	$8.43750\;\times \;10^{11}$
BWO	4.250741	$4.00\;\times \;10^{12}$	$1.14286\;\times \;10^{12}$	$1.85164\;\times \;10^{12}$	6.284603	$4.00\;\times \;10^{12}$

**Table 22:** Statistical results on the Tension/Compression Spring design problem (dim = 3).

Algorithm	Best	Worst	Mean	Std	Median	IQR
SFOA	0.00890241	0.00890241	0.00890241	$1.57222\;\times \;10^{-12}$	0.00890241	$1.22996\;\times \;10^{-12}$
SFOA_FDB_Case_1_163	0.00890241	0.00890241	0.00890241	$3.38033\;\times \;10^{-12}$	0.00890241	$1.34788\;\times \;10^{-13}$
SFOA_dFDB_Case_1_205	0.00890241	0.00890241	0.00890241	$1.70650\;\times \;10^{-12}$	0.00890241	$6.11754\;\times \;10^{-13}$
FDA	0.011147805	0.062237488	0.018681723	0.011877186	0.014117725	0.008539235
GEO	0.009149058	0.012937545	0.009987199	0.000802199	0.009767345	0.000638322
GJO	0.009150978	0.029557755	0.016055459	0.005828325	0.014627132	0.003764535
SAO	0.022560331	$9.64997\;\times \;10^{11}$	$2.30528\;\times \;10^{11}$	$3.32777\;\times \;10^{11}$	$1.76915\;\times \;10^{10}$	$3.39783\;\times \;10^{11}$
RSA	0.013232265	$1.69550\;\times \;10^{11}$	$8.07379\;\times \;10^{9}$	$3.69987\;\times \;10^{10}$	0.016559424	0.032578668
SOS	0.009062206	0.012117376	0.009628249	0.000623303	0.009561878	0.000429216
MRFO	0.008956059	0.015098274	0.009981511	0.001732336	0.009191100	0.000840002
KOA	0.009024682	0.010190052	0.009449794	0.000382900	0.009328742	0.000528040
CO	0.008986522	0.015567315	0.011347821	0.001773201	0.011169355	0.002512989
DAOA	0.013225448	0.014299234	0.013739248	0.000367498	0.013734296	0.000713628
BWO	0.010185486	$5.44094\;\times \;10^{10}$	$5.74125\;\times \;10^{9}$	$1.51794\;\times \;10^{10}$	0.016934601	0.007370381

**Table 23:** Statistical results on the Pressure Vessel design problem (dim = 4).

Algorithm	Best	Worst	Mean	Std	Median	IQR
SFOA	5885.332888	5885.345512	5885.334306	0.002643	5885.333583	0.001020
SFOA_FDB_Case_1_163	5885.332778	5885.333069	5885.332855	$7.41592\;\times \;10^{-5}$	5885.332841	$9.56119\;\times \;10^{-5}$
SFOA_dFDB_Case_1_205	5885.332775	5885.334157	5885.332980	0.000317	5885.332874	0.000212
FDA	8365.448092	18,661.580280	12,763.299960	2653.477203	11,720.928120	4168.076810
GEO	8088.230023	25,768.160110	15,062.760280	4324.659178	15,735.042350	5116.371510
GJO	7915.547660	37,867.392500	19,753.345580	8883.552047	16,760.415580	12,241.950110
SAO	14,129.609890	30,672.607430	21,810.887390	3615.610522	20,574.998820	3930.149305
RSA	9939.653573	29,469.750020	16,512.243240	5086.323369	15,502.592900	7794.397137
SOS	6304.938078	9442.373025	7636.694748	765.097565	7557.144942	1205.072694
MRFO	6119.948689	9378.911646	7253.957334	952.415591	6983.025207	1309.053053
KOA	6052.201334	8392.497681	6960.059604	539.978460	6867.657712	525.280428
CO	5940.354914	7303.058484	6586.594497	408.522049	6530.386452	490.132707
DAOA	9943.873072	58,584.652530	22,140.794380	11,147.196780	18,274.462310	10,090.697350
BWO	8026.494136	19,931.220290	14,000.934770	3642.302131	13,918.422210	5707.478574

**Table 24:** Statistical results on the Blending–Pooling problem, D=38, over 21 independent runs.

Algorithm	Best	Worst	Mean	Std	Median	IQR
SFOA	5.101887 $\times 10^{11}$	$7.90667\times 10^{13}$	$2.23459\times 10^{13}$	$2.74400\times 10^{13}$	$9.90175\times 10^{12}$	$3.93019\times 10^{13}$
SFOA_FDB_Case_1_163	$4.999994\times 10^{11}$	$8.00000\times 10^{13}$	$1.74569\times 10^{13}$	$2.08799\times 10^{13}$	$3.870369\times 10^{12}$	$2.409693\times 10^{13}$
SFOA_dFDB_Case_1_205	$4.999994\times 10^{11}$	$8.00000\times 10^{13}$	$1.48282\times 10^{13}$	$1.89850\times 10^{13}$	$5.29469\times 10^{12}$	$2.001039\times 10^{13}$
FDA	$2.59440\times 10^{13}$	$8.38698\times 10^{13}$	$6.08194\times 10^{13}$	$1.65682\times 10^{13}$	$5.83924\times 10^{13}$	$2.28339\times 10^{13}$
GEO	$1.65340\times 10^{14}$	$3.98213\times 10^{14}$	$2.54683\times 10^{14}$	$6.03472\times 10^{13}$	$2.45720\times 10^{14}$	$7.35022\times 10^{13}$
GJO	$9.25437\times 10^{13}$	$2.28995\times 10^{14}$	$1.40409\times 10^{14}$	$3.47216\times 10^{13}$	$1.33936\times 10^{14}$	$4.25640\times 10^{13}$
SAO	$5.08630\times 10^{14}$	$8.36753\times 10^{14}$	$7.17668\times 10^{14}$	$9.11154\times 10^{13}$	$7.35452\times 10^{14}$	$1.50496\times 10^{14}$
RSA	$8.60000\times 10^{13}$	$1.98631\times 10^{14}$	$1.01993\times 10^{14}$	$3.54424\times 10^{13}$	$8.60000\times 10^{13}$	$2.32752\times 10^{11}$
SOS	$2.33820\times 10^{13}$	$8.04830\times 10^{13}$	$4.80767\times 10^{13}$	$1.44263\times 10^{13}$	$4.52818\times 10^{13}$	$8.13297\times 10^{12}$
MRFO	$3.54265\times 10^{13}$	$8.47738\times 10^{13}$	$7.30793\times 10^{13}$	$1.65177\times 10^{13}$	$8.14623\times 10^{13}$	$7.96667\times 10^{12}$
KOA	$1.32209\times 10^{13}$	$7.99986\times 10^{13}$	$3.62867\times 10^{13}$	$1.72446\times 10^{13}$	$3.37357\times 10^{13}$	$1.60076\times 10^{13}$
CO	$8.33194\times 10^{12}$	$4.49850\times 10^{13}$	$3.11215\times 10^{13}$	$9.89173\times 10^{12}$	$2.89954\times 10^{13}$	$1.36986\times 10^{13}$
DAOA	$2.66000\times 10^{14}$	$2.66000\times 10^{14}$	$2.66000\times 10^{14}$	$2.41534\times 10^{5}$	$2.66000\times 10^{14}$	$0.00000\times 10^{0}$
BWO	$8.30358\times 10^{13}$	$8.59912\times 10^{13}$	$8.44456\times 10^{13}$	$7.09120\times 10^{11}$	$8.43908\times 10^{13}$	$6.88374\times 10^{11}$

**Table 25:** Best design variables obtained by FDBSFOA and dFDBSFOA for the four-stage gear box problem.

Algorithm/Variable Group	Best Design Variables
FDBSFOA	Best objective = 36.5652
$x_{1}–x_{8}$	[19.8782,51.2983,17.1112,34.3206,22.3076,39.7709,23.3739,48.6870]
$x_{9}–x_{12}$	[1.3970,0.6557,1.3449,0.9469]
$x_{13}–x_{22}$	[3.0330,4.9148,3.1870,5.4844,4.3539,2.9251,6.1445,5.9401,4.5592,6.1849]
dFDBSFOA	Best objective = 36.4779
$x_{1}–x_{8}$	[22.1569,43.7370,23.3588,56.7585,19.4262,37.8692,16.8080,33.6064]
$x_{9}–x_{12}$	[1.4123,1.0878,0.5364,1.3864]
$x_{13}–x_{22}$	[1.5172,5.4969,4.3494,5.4045,4.9078,6.1322,6.3241,3.3398,6.2112,6.3540]

**Table 26:** Four-Stage Gear problem, D=22, over 21 independent runs.

Algorithm	Best	Worst	Mean	Std	Median	IQR
SFOA	4.31902 $\times 10^{1}$	$1.62189\times 10^{7}$	$2.11898\times 10^{6}$	$4.46389\times 10^{6}$	$1.29689\times 10^{5}$	$1.20973\times 10^{6}$
SFOA_FDB_Case_1_163	$3.85889\times 10^{1}$	$1.20356\times 10^{7}$	$1.56116\times 10^{6}$	$3.41335\times 10^{6}$	$6.46431\times 10^{1}$	$1.19257\times 10^{6}$
SFOA_dFDB_Case_1_205	$3.62515\times 10^{1}$	$1.91201\times 10^{6}$	$3.88727\times 10^{5}$	$6.13619\times 10^{5}$	$6.21279\times 10^{1}$	$1.10436\times 10^{6}$
FDA	$8.05387\times 10^{7}$	$3.47937\times 10^{8}$	$2.09541\times 10^{8}$	$6.26266\times 10^{7}$	$1.99290\times 10^{8}$	$9.16726\times 10^{7}$
GEO	$4.73407\times 10^{1}$	$2.96042\times 10^{7}$	$7.33825\times 10^{6}$	$8.45020\times 10^{6}$	$5.92710\times 10^{6}$	$1.07404\times 10^{7}$
GJO	$8.61926\times 10^{6}$	$1.06293\times 10^{9}$	$1.05587\times 10^{8}$	$1.83545\times 10^{8}$	$5.79564\times 10^{7}$	$4.75726\times 10^{7}$
SAO	$5.57408\times 10^{8}$	$6.68506\times 10^{9}$	$3.04094\times 10^{9}$	$1.48530\times 10^{9}$	$3.06182\times 10^{9}$	$2.15024\times 10^{9}$
RSA	$8.97189\times 10^{7}$	$1.10633\times 10^{9}$	$2.38063\times 10^{8}$	$2.20358\times 10^{8}$	$1.56141\times 10^{8}$	$1.45130\times 10^{8}$
SOS	$3.10906\times 10^{6}$	$9.43593\times 10^{7}$	$4.01903\times 10^{7}$	$2.65212\times 10^{7}$	$3.53964\times 10^{7}$	$3.89853\times 10^{7}$
MRFO	$2.28887\times 10^{7}$	$3.30964\times 10^{8}$	$1.49319\times 10^{8}$	$7.30464\times 10^{7}$	$1.38254\times 10^{8}$	$8.70503\times 10^{7}$
KOA	$4.66962\times 10^{1}$	$2.21585\times 10^{7}$	$6.64876\times 10^{6}$	$6.47933\times 10^{6}$	$4.85846\times 10^{6}$	$8.66367\times 10^{6}$
CO	$5.45274\times 10^{1}$	$2.10813\times 10^{7}$	$3.24575\times 10^{6}$	$4.93668\times 10^{6}$	$1.34479\times 10^{6}$	$3.99426\times 10^{6}$
DAOA	$1.98573\times 10^{8}$	$1.60534\times 10^{9}$	$7.51883\times 10^{8}$	$3.52131\times 10^{8}$	$6.98273\times 10^{8}$	$4.32831\times 10^{8}$
BWO	$4.64563\times 10^{7}$	$3.25915\times 10^{8}$	$1.38085\times 10^{8}$	$6.42111\times 10^{7}$	$1.18701\times 10^{8}$	$8.88021\times 10^{7}$

## Data Availability

The MATLAB codes and supporting files used in this study are publicly available at MathWorks File Exchange: https://www.mathworks.com/matlabcentral/fileexchange/182481-dfdb-sfoa-an-improved-starfish-optimization-algorithm?s_tid=srchtitle_site_search_2_sfoa (accessed on 5 November 2025).
